# Immune-related gene expression links anxiety, depression, and pain outcomes in adolescence

**DOI:** 10.1038/s43856-026-01960-5

**Published:** 2026-10-16

**Authors:** Anh Cong Tuan Le, Tobias Banaschewski, Arun L. W. Bokde, Rüdiger Brühl, Sylvane Desrivières, Hugh Garavan, Penny Gowland, Antoine Grigis, Andreas Heinz, Jean-Luc Martinot, Marie-Laure Paillère Martinot, Eric Artiges, Dimitri Papadopoulos Orfanos, Luise Poustka, Michael N. Smolka, Nathalie Holz, Nilakshi Vaidya, Henrik Walter, Robert Whelan, Paul Wirsching, Gunter Schumann, Herta Flor, Frauke Nees, IMAGEN Consortium, Tobias Banaschewski, Arun L. W. Bokde, Rüdiger Brühl, Sylvane Desrivières, Hugh Garavan, Penny Gowland, Antoine Grigis, Andreas Heinz, Jean-Luc Martinot, Marie-Laure Paillère Martinot, Eric Artiges, Luise Poustka, Michael N. Smolka, Nathalie Holz, Nilakshi Vaidya, Henrik Walter, Robert Whelan, Paul Wirsching, Gunter Schumann, Herta Flor, Frauke Nees, Dimitri Papadopoulos Orfanos

**Affiliations:** 1 Institute of Medical Psychology and Medical Sociology, University Medical Center Schleswig-Holstein, Kiel University, Kiel, Germany; 2 Department of Neuropsychology and Resilience Research, Research Group Learning and Brain Plasticity in Mental Disorders, Central Institute of Mental Health, Medical Faculty Mannheim, Heidelberg University, Mannheim, Germany; 3 Life and Health Sciences Research Institute (ICVS), School of Medicine, University of Minho, Braga, Portugal; 4 ICVS/3B’s - PT Government Associate Laboratory, Braga/Guimarães, Portugal; 5 Department of Health Science and Technology, Center for Neuroplasticity and Pain (CNAP), Aalborg University, Gistrup, Denmark; 6 Department of Child and Adolescent Psychiatry and Psychotherapy, Central Institute of Mental Health, Medical Faculty Mannheim, Heidelberg University, Mannheim, Germany; 7 German Center for Mental Health (DZPG), Partner Site Mannheim-Heidelberg-Ulm, Mannheim, Germany; 8 Discipline of Psychiatry, School of Medicine and Trinity College Institute of Neuroscience, Trinity College Dublin, Dublin, Ireland; 9 Physikalisch-Technische Bundesanstalt (PTB), Braunschweig and Berlin, Berlin, Germany; 10 Social, Genetic and Developmental Psychiatry Centre, Institute of Psychiatry, Psychology & Neuroscience, King’s College London, London, UK; 11 Departments of Psychiatry and Psychology, University of Vermont, Burlington, VT USA; 12 Sir Peter Mansfield Imaging Centre School of Physics and Astronomy, University of Nottingham, University Park, Nottingham, UK; 13 NeuroSpin, CEA, Université Paris-Saclay, Gif-sur-Yvette, France; 14 Department of Psychiatry and Psychotherapy, University of Tübingen, Tübingen, Germany; 15 German Center for Mental Health (DZPG), Site Tübingen, Germany; 16 Institut National de la Santé et de la Recherce Médicale, INSERM U A10 “Trajectoires développementales & psychiatrie”, University Paris-Saclay, Ecole Normale Supérieure Paris-Saclay, CNRS; Centre Borelli, Gif-sur-Yvette, France; 17 AP-HP. Sorbonne Université, Department of Child and Adolescent Psychiatry, Pitié-Salpêtrière Hospital, Paris, France; 18 Psychiatry Department, EPS Barthélémy Durand, Etampes, France; 19 Department of Psychology, School of Social Sciences, University of Mannheim, 68131 Mannheim, Germany; 20 Department of Child and Adolescent Psychiatry, Center for Psychosocial Medicine, University Hospital Heidelberg, Heidelberg, Germany; 21 Department of Psychiatry and Psychotherapy, Technische Universität Dresden, Dresden, Germany; 22 Centre for Population Neuroscience and Stratified Medicine (PONS), Department of Psychiatry and Psychotherapy, Charité Universitätsmedizin Berlin, Berlin, Germany; 23 Department of Psychiatry and Psychotherapy CCM, Charité – Universitätsmedizin Berlin, corporate member of Freie Universität Berlin, Humboldt-Universität zu Berlin, and Berlin Institute of Health, Berlin, Germany; 24 School of Psychology and Global Brain Health Institute, Trinity College Dublin, Dublin, Ireland; 25 Centre for Population Neuroscience and Precision Medicine (PONS), Institute for Science and Technology of Brain-inspired Intelligence (ISTBI), Fudan University, Shanghai, China; 26 German Center for Mental Health (DZPG), Site Berlin-, Potsdam, Germany; 27 Institute of Medical Psychology, LMU Medizin, Ludwig-Maximilians-Universität, Munich, Germany

**Keywords:** Predictive markers, Genetic predisposition to disease

## Abstract

**Background:**

Chronic pain and internalising disorders such as anxiety and depression frequently co-occur during adolescence. Although prospective associations between internalising symptoms and later pain have been reported previously, the biological mechanisms underlying individual differences in this relationship remain poorly understood. Immune- and oxidative stress-related pathways, including the *NRF2* gene, may contribute to such variability. This study investigated whether polymorphisms in the *NRF2* gene (rs6721961 and rs35652124) are related to the association between anxiety and depression symptoms at age 14-15 and pain complaints two years later at age 16-17.

**Methods:**

Data were drawn from the IMAGEN longitudinal study (*N* = 724). Anxiety and depression symptoms were assessed using the Development and Well-Being Assessment (DAWBA), and pain complaints were measured via the Children’s Somatization Inventory (CSI). Genotyping was conducted for *NRF2* SNPs rs6721961 and rs35652124. Regression analyses tested main effects, followed by genotype-stratified analyses.

**Results:**

Consistent with previous literature, higher levels of anxiety and depression at age 14-15 predicted increased pain complaints at age 16-17. Carriers of the TG/TT genotype of rs6721961 had significantly lower anxiety scores and reported fewer pain complaints compared to those carrying the GG genotype of rs6721961. Notably, the association between anxiety and later pain was significant only among individuals with the GG genotype.

**Conclusion:**

While replicating established associations between adolescent early internalising symptoms and later pain, this study suggests that genetic variation in NRF2 is related to the anxiety-pain association. Specifically, rs6721961 is associated with differences in susceptibility to pain among adolescents with elevated anxiety symptoms.

## Plain Language Summary

Anxiety and depression symptoms during adolescence are associated with greater pain complaints later in life, but the biological mechanisms underlying this relationship remain unclear. Emerging evidence suggests that NRF2, a protein that helps regulate the body’s response to inflammation and oxidative stress, may play an important role. Using data from a longitudinal study, in which symptoms of anxiety, depression and pain were assessed, we found that adolescents with higher levels of anxiety and depression at age 14 reported more pain complaints at age 16. We also found that a common genetic variation in the *NRF2* gene (rs6721961) was associated with differences in vulnerability to pain among adolescents with elevated anxiety symptoms. These findings support further research into the role of NRF2-related genetic variation in pain vulnerability and may improve our understanding of early risk pathways in young people experiencing psychological symptoms.

## Abstract (web summary)

Le et al. use longitudinal data to examine whether polymorphisms in the NRF2 gene are associated with affective symptoms and pain. They find that higher levels of anxiety and depression at age 14 predicted increased pain complaints at age 16 and the NRF2 rs6721961 polymorphism is associated with differential vulnerability to pain.

## Introduction

Adolescence is a critical developmental period marked by substantial changes in brain structure and function, particularly in regions involved in affective processing, cognitive control, and pain modulation^1,2^. During this time, individuals become increasingly vulnerable to both internalising symptoms (e.g., anxiety and depression) and somatic complaints, including recurrent and chronic pain^3,4^. Epidemiological studies indicate a high prevalence of pain among adolescents, with over 60% reporting headaches annually and approximately one-third affected by musculoskeletal or chronic pain conditions^5,6^.

Importantly, pain in adolescence is not merely a physical phenomenon - it is closely linked to psychological stress and affective dysregulation^7^. Co-occurrence of anxiety, depression, and pain is well-documented, and prospective studies suggest that internalising symptoms may increase the risk of later pain complaints. However, not all adolescents with elevated anxiety or depression develop persistent pain, indicating substantial interindividual variability in vulnerability. The biological mechanisms underlying these individual differences remain poorly understood.

One biological pathway increasingly implicated in both affective and pain disorders is the immune system, particularly neuroimmune interactions that shape brain development during adolescence^8^. For example, elevated levels of pro-inflammatory cytokines such as IL-6 and TNF-α have been associated with both anxiety and depression^9,10^, while interventions targeting inflammatory pathways have shown therapeutic effects in mood disorders^11^. Similarly, immune dysregulation is reported to contribute to pain chronification via glial activation, cytokine release, and sensitisation of central nociceptive pathways^12,13^. Collectively, this evidence suggests that immune-related mechanisms may influence susceptibility to both internalising symptoms and pain, as well as the relationship between them.

A candidate molecular regulator in this context is nuclear factor erythroid 2-related factor 2 (NRF2) - a transcription factor that orchestrates antioxidant and anti-inflammatory responses^14^. Activation of NRF2 has been shown to mitigate both pain behaviours and affective symptoms in animal models^15,16^, while our recent work suggests that NRF2 may contribute to individual susceptibility and resilience to chronic pain and its affective comorbidity^17^. At the genetic level, promoter-region polymorphisms such as rs6721961 and rs35652124, which are linked to different regulatory motifs, have been associated with altered *NRF2* expression and transcriptional activity, potentially influencing inflammatory and oxidative stress responses^18–21^. These variants, therefore, represent plausible candidates for moderating the link between internalising symptoms and later pain outcomes. Despite accumulating evidence for prospective associations between internalising symptoms and pain, and growing evidence implicating NRF2 in both affective and pain-related processes, it remains unknown whether genetic variation in NRF2 is associated with individual differences in the strength of this association during adolescence.

The present study addresses this gap using data from a large adolescent cohort. We investigated whether two common *NRF2* polymorphisms (rs6721961 and rs35652124) are related to the association between anxiety and depression symptoms at age 14–15 and pain complaints two years later at age 16–17. Specifically, we were interested in whether the genotype groups differ in this association. Based on previous literature, we expected higher internalising symptoms to be associated with greater pain complaints at follow-up and hypothesized that NRF2 genetic variation would be related to individual differences in susceptibility to this affect-pain relationship.

Consistent with previous literature, we found that greater levels of anxiety and depression at age 14–15 are associated with increased pain complaints at age 16–17. Individuals carrying the TG/TT genotype of rs6721961 report significantly lower anxiety scores and fewer pain complaints than GG genotype carriers. Notably, in the genotype-stratified analyses, the association between early anxiety and later pain reaches statistical significance among individuals with the GG genotype, but not among those with the TG/TT genotype.

## Methods

### Participants

We leveraged data from the IMAGEN study (“Reinforcement-related behaviour in normal brain function and psychopathology”; grant LSHM-CT-2007-037286; data collection started in 2008)^22,23^, in which healthy adolescents were recruited from the general population via school visits, flyers, and registration offices in Germany, the United Kingdom, Ireland, and France. Exclusion criteria for study participation were: contraindications for magnetic resonance imaging (MRI) examinations, pregnancy, severe medical conditions, and previous head trauma with unconsciousness. For the present analyses, a subset of participants with complete baseline (BL) (age 14–15) and follow-up 1 (FU1) (age 16–17) data was used (*N* = 724) (Table 1).

**Table 1:** Participants’ characteristics

Characteristics	Included sample	Excluded sample	Statistics
*N*	724	1461	
Gender (m/f)	312/412	760/701	*χ*^2^(1) = 15.076, *p* = 1.033e-4
Age BL	14.439 (0.380)	14.424 (0.394)	*t* = −0.839, *p* = 0.4017
Anxiety BL	1.233 (0.549)	1.243 (0.562)	*χ*^2^(3) = 0.305, *p* = 0.959
Depression BL	0.928 (1.006)	0.962 (1.033)	*χ*^2^(4) = 1.339, *p* = 0.8551
Age FU1	16.097 (0.085)	16.101 (0.086) *N* = 495	*t* = 0.831, *p* = 0.4061
Pain sum score FU1	3.301 (3.308)	3.212 (3.107) *N* = 495	*χ*^2^(19) = 21.448, *p* = 0.3126
Participation rate per center; *n* (%)	London 81 (11.187) Nottingham 92 (12.707) Dublin 63 (8.702) Berlin 84 (11.602) Hamburg 106 (14.641) Mannheim 96 (13.260) Paris 80 (11.050) Dresden 122 (16.851)	190 (13.004) 269 (18.412) 170 (11.636) 185 (12.663) 159 (10.883) 169 (11.567) 182 (12.457) 137 (9.377)	*χ*^2^(7) = 45.567, *p* = 1.061e-07

The study was approved by the local ethics committees, including the institutional ethics committee of King’s College London (PNM/10/11-126), University of Nottingham (D/11/2007), Trinity College Dublin (SPREC092007-01), Technische Universitat Dresden (EK 235092007), Commissariat a l’Energie Atomique et aux Energies Alternatives, INSERM (2007-A00778-45), University Medical Center at the University of Hamburg (M-191/07) and in Germany at medical ethics committee of the University of Heidelberg (2007-024N-MA) in accordance with the Declaration of Helsinki. After a complete study description, written informed consent was obtained from both adolescents and their parents.

### Assessment of anxiety and depression

The Development and Well-Being Assessment (DAWBA)^24^ was utilized to assess major depressive disorder (MDD) and generalized anxiety disorder (GAD), with definite symptoms obtained by structured questions to the child and parent. The answers to those questions were fed into a computerized diagnostic algorithm, which predicts the likelihood that an experienced clinical rater would assign the child operationalized DSM-IV and ICD-10 diagnoses. The computer prediction provides output as a 6-point scale, ranging from less than 0.1% likely to more than 70% likely. Data on GAD and MDD were used at BL.

### Assessment of pain symptoms

The participants completed the Children Somatization Inventory (CSI)^25^ to assess the severity of somatic symptoms, including pain. The CSI consists of 35 items, including the somatic symptoms defined by DSM-III-R (American Psychiatric Association, 1987), the Hopkins Symptom Checklist (HSCL)^26^, and additional symptoms common in functional gastrointestinal disorders. The response format is a 5-point scale ranging from “*not at all*” (0) to “*a whole lot*” (4), which results in a range of sum scores from 0 to 140, and the standard period for symptom reports is 2 weeks. Items that are related to pain and motor weakness symptoms were selected based on ref. ^27^. Participants completed the CSI online from home using Psytools software (Delosis Ltd, London, United Kingdom). In this study, we included data on pain symptoms at FU1.

### DNA extraction, genotyping, and quality control

Whole blood samples (~10 mL) were collected and stored in BD Vacutainer EDTA tubes (Becton, Dickinson and Company, Oxford, United Kingdom). DNA extraction was performed using Gentra Puregene Blood Kit (QIAGEN Inc., Valencia, CA), while genotyping was done at 582,982 markers within the context of the IMAGEN study^22^ using the Illumina HumanHap610 Genotyping BeadChip (Illumina, San Diego, CA). Single nucleotide polymorphisms were excluded if call rates were below 98%, minor allele frequency under 1%, or significant deviation from Hardy–Weinberg equilibrium (*P* ≤ 1 × 10^−^^4^). Additionally, individuals with excessive missing genotypes (failure rate > 2%), outlying heterozygosity (more than three standard deviations from the mean), or an ambiguous sex code were also excluded. Before the following analysis, closely related individuals with an identity-by-descent greater than 0.1875 were excluded. Population stratification for the GWAS was examined by principal component analysis with EIGENSTRAT software^28^ and the four HapMap II populations as references. Additionally, individuals with divergent ancestry (from CEU [Utah residents of northern and western European ancestry]) were also excluded^29^.

For this study, we report data on 589 GG genotype carriers of rs6721961, 130 TG, and 5 TT genotype carriers. Due to a limited number of TT alleles, TG and TT will be merged (*N* = 135). Meanwhile, the SNP rs35652124 consists of 337 TT, 312 CT, and 75 CC. Please refer to Table 2 for more details on the SNPs. The present hypotheses were not pre-registered. However, the selection of NRF2 and its SNPs was guided by a priori biological considerations and based on the established role of NRF2 in oxidative stress and immune response regulation implicated in both pain and affective symptoms.

**Table 2:** Details on SNPs rs6721961 and rs35652124 of the human *NRF2*

SNP	Position	Localization	Allele (minor/major)
rs6721961	177265309	GRCh38, Chr 2	T/G
rs35652124	177265345	GRCh38, Chr 2	C/T

### Statistics and reproducibility

#### Association between anxiety/depression and pain complaints during adolescence

For the prediction of pain complaints, ordinary least-squares regression was used, with anxiety/depression measured at BL as a regressor for painful symptoms at FU1. Anxiety and depression scores were treated as continuous variables in the regression models, while sex and centre sites were added as covariates - Eq. (1–2).

1 $${{pain}}\,{{sum}}\,{{score}}\,\left({{FU}}1\right)\sim\,{{GAD}}\,\left({{BL}}\right)+{{sex}}+{{centre}}\,{{sites}}$$

2 $${{{\mathrm{pain}}}}\,{{{\mathrm{sum}}}}\,{{{\mathrm{score}}}}\,\left({{{\mathrm{FU}}}}1\right)\sim\,{{MDD}}\,\left({{{\mathrm{BL}}}}\right)+{{{\mathrm{sex}}}}+\,{{{\mathrm{centre}}}}\,{{{\mathrm{sites}}}}$$

#### The contributions of SNPs rs35652124 and rs6721961 to the associations

The prediction models were tested in the whole group and separately for the allele groups of rs6721961 and rs35652124. Moreover, we analyzed possible differences in pain complaints and early-in-life anxiety/depression symptoms between the allele groups using either analysis of variance (ANOVA) or independent *t*-tests if the data met parametric assumptions. Otherwise, the Kruskal–Wallis test or the Mann–Whitney U test was employed.

#### Statistical significance, multiple comparison correction, and identified outliers

All tests were performed with a significance level of *α* = 0.05, two-tailed. Data were presented as mean (SD). Benjamini–Hochberg correction was applied for multiple comparisons. In this study, significant outliers were defined using Cook’s distance of 4/(*n* – *k* – 1), where n is the sample size and k is the number of independent variables. Analyses were performed in Python 3.9.6 and R 4.6.0.

To access the robustness and specificity of the outcome, analyses were also conducted using *NRF2* rs2886161 as a negative control, supplemented with permutation testing (Tables S1–S3 and Fig. S1). Compared to the two main SNPs, the negative control is located in a different region (Intron 1) in the NRF2 gene and has few prior studies linking it with pain and comorbidities^30^.

## Results

### Genotype differences and genotype-stratified analyses

#### Pain complaints and *NRF2* (rs6721961 and rs35652124)

**Fig. 1:**
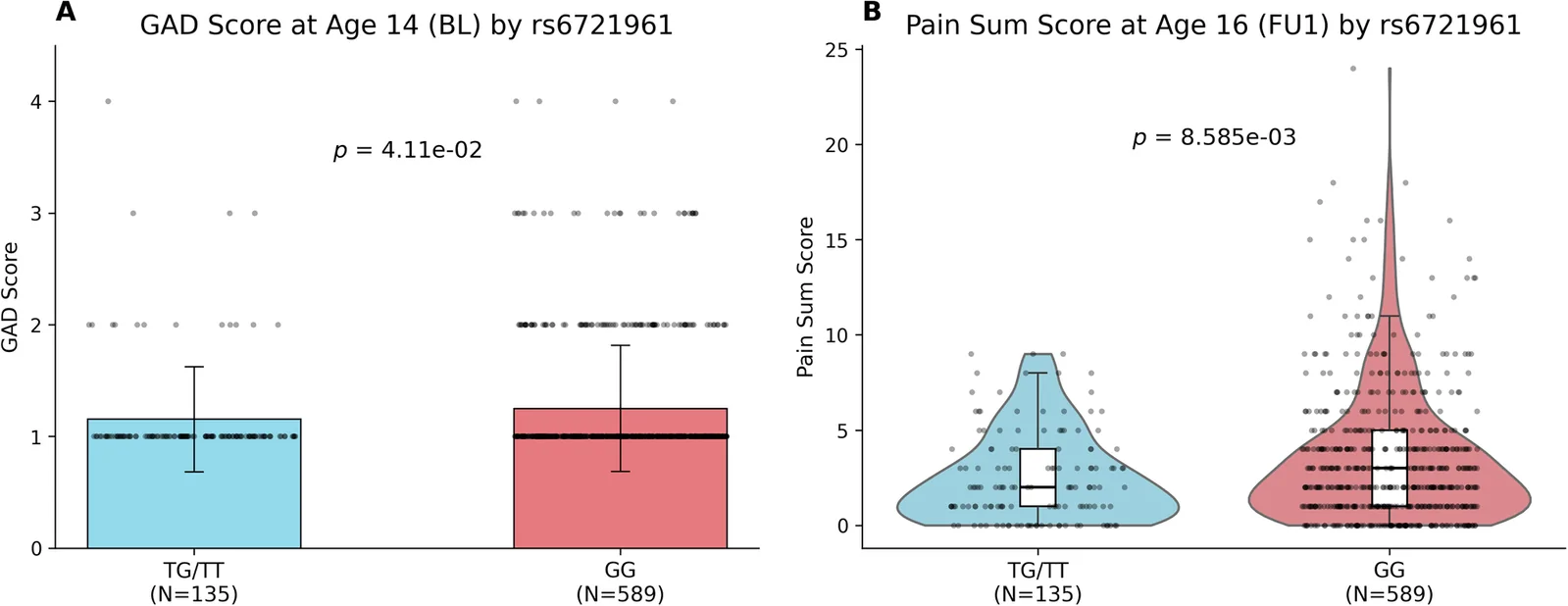
Anxiety score at age 14 and pain complaints at age 16 between rs6721961 *NRF2* variants. The TG/TT allele carriers presented (**A**) a lower anxiety score at age 14 and (**B**) fewer pain complaints at age 16 than those possessing the GG genotype. **A** Bars represent mean (SD) anxiety score. **B** Violin plots with an embedded boxplot (median, interquartile range). Mann–Whitney *U* tests (two-sided) were conducted. GAD generalized anxiety disorder, BL baseline, FU1 follow-up 1.

A Mann–Whitney *U* test revealed significant differences in total pain complaint scores at age 16–17 between genotype groups of rs6721961 (*U* = 34,052, *p* = 8.585e-03). Individuals carrying the TG/TT genotype reported fewer pain symptoms (*M* = 2.53 (2.36)) compared to those with the GG genotype (*M* = 3.48 (3.47)) (see Table 3, Fig. 1).

**Table 3:** Characteristics of CSI pain-related items for rs6721961

Pain/weakness CSI items	TG/TT	GG	statistics (U)	*p* value raw	*p* value adjusted
Pain heart chest	0.193 (0.414)	0.275 (0.565)	37961	2.529403e-01	3.161753e-01
Sore muscles	0.519 (0.621)	0.662 (0.782)	36752	1.288146e-01	2.442074e-01
Trouble getting breath	0.141 (0.476)	0.197 (0.497)	37618	1.131528e-01	2.442074e-01
Numbness or tingling	0.23 (0.472)	0.333 (0.545)	36172	3.654053e-02	1.218018e-01
Weakness	0.474 (0.584)	0.545 (0.714)	38744	5.994062e-01	6.660069e-01
Heavy feelings in arms legs	0.296 (0.519)	0.406 (0.657)	37176	1.465244e-01	2.442074e-01
Heart beating too fast	0.148 (0.432)	0.209 (0.501)	37944	1.955837e-01	2.794053e-01
Difficulty swallowing	0.141 (0.408)	0.153 (0.433)	39412.5	7.845590e-01	7.845590e-01
Pain knees elbow joints	0.215 (0.463)	0.365 (0.634)	35755	1.898543e-02	9.492714e-02
Pain arms legs	0.17 (0.397)	0.334 (0.599)	34913.5	3.734203e-03	3.734203e-02
Pain sum score	2.526 (2.359)	3.479 (3.466)	34052	8.585498e-03	8.585498e-03

Notably, this difference appeared to be pronounced for pain types resembling neuropathic symptoms, including pain in arms/legs (*p* = 3.734e-03), numbness/tingling (*p* = 3.654e-02), and, to a lesser extent, joint pain (*p* = 1.899e-02). However, only pain in arms/legs survived multiple testing corrections (Table 3).

A permutation test (10,000 permutations) showed that the observed difference in mean pain sum score between rs6721961 genotypes was unlikely under the null distribution (empirical *p* = 0.0022). Meanwhile, the total pain scores were not significantly different between alleles of the negative control rs2886161 (Tables S1–S3 and Fig. S1).

For rs35652124, no significant differences in total pain complaints scores were observed across genotypes (Kruskal–Wallis test, *p* > 0.05) (Table 4).

**Table 4:** Characteristics of CSI pain-related items for rs35652124

Pain/weakness CSI items	CC	CT	TT	Statistic (H)	*p* value raw	*p* value adjusted
Pain heart chest	0.293 (0.564)	0.288 (0.532)	0.226 (0.542)	5.140227533	7.652684e-02	3.826342e-01
Sore muscles	0.693 (0.771)	0.615 (0.769)	0.641 (0.743)	1.113659316	5.730229e-01	9.650210e-01
Trouble getting breath	0.333 (0.622)	0.17 (0.474)	0.169 (0.474)	9.251365646	9.796963e-03	9.796963e-02
Numbness or tingling	0.293 (0.514)	0.308 (0.515)	0.323 (0.556)	0.099157027	9.516304e-01	9.650210e-01
Weakness	0.56 (0.721)	0.564 (0.701)	0.496 (0.678)	1.990668212	3.695999e-01	9.650210e-01
Heavy feelings in arms legs	0.36 (0.584)	0.404 (0.664)	0.374 (0.619)	0.139076136	9.328246e-01	9.650210e-01
Heart beating too fast	0.2 (0.493)	0.224 (0.539)	0.172 (0.436)	1.044758522	5.931077e-01	9.650210e-01
Difficulty swallowing	0.173 (0.554)	0.147 (0.414)	0.148 (0.41)	0.160113565	9.230639e-01	9.650210e-01
Pain knees elbows joints	0.267 (0.553)	0.337 (0.594)	0.353 (0.634)	1.388783269	4.993782e-01	9.650210e-01
Pain arms legs	0.333 (0.644)	0.288 (0.532)	0.312 (0.588)	0.071210891	9.650210e-01	9.650210e-01
Pain sum score	3.507 (3.696)	3.346 (3.271)	3.214 (3.258)	0.439114477	8.028742e-01	8.028742e-01

Significant sex differences were also observed: female adolescents reported more pain (*M* = 3.72 (3.59)) than males (*M* = 2.75 (2.80)) (*p* = 1.083e-04). There was no association between sex and rs6721961 genotype distribution (*χ*² = 0.017, *p* > 0.05).

#### Anxiety, depression, and *NRF2* genotypes

At age 14–15, carriers of the TG/TT genotype of rs6721961 reported significantly lower anxiety symptoms than GG carriers (*U* = 36,769.5, *p* = 4.11e-02), with no significant differences in depression scores (*U* = 39,225, *p* > 0.05) (Table 5 and Fig. 1).

**Table 5:** Anxiety, depression, and *NRF2* polymorphisms

Genotypes		Anxiety BL	Depression BL	*N*
rs6721961	TG/TT	1.156 (0.471)	0.881 (0.939)	135
	GG	1.251 (0.564)	0.939 (1.022)	589
rs35652124	CC	1.200 (0.465)	0.987 (0.908)	75
	CT	1.228 (0.516)	0.910 (1.010)	312
	TT	1.246 (0.594)	0.932 (1.026)	337

Again, rs35652124 was not associated with differences in either anxiety or depression (*p* > 0.05).

Sex differences emerged in both anxiety (M: 1.09 (0.33); F: 1.34 ± 0.65, *p* = 2.653e-10) and depression (M: 0.71 (0.82); F: 1.09 (1.10), *p* = 1.686e-07), but sex did not moderate the relationship between internalising symptoms and later pain (*p* > 0.05).

#### Anxiety-pain association stratified by *NRF2* (rs6721961) genotype

To assess whether the association between early anxiety symptoms and later pain complaints differed across rs6721961 genotype groups, stratified regression analyses were performed:

- Among GG genotype carriers, anxiety symptoms at age 14–15 significantly predicted pain complaints at age 16–17 (*β* = 0.238, 95% CI: 0.156–0.320, *p* = 1.87e-8), explaining 7.6% of the variance (*R*² = 0.076, *p* = 1.17e-08).
- Among TG/TT carriers, the same association was non-significant (*p* > 0.05) (Supplementary Data 1). No formal anxiety-by-genotype interaction was demonstrated.

**Fig. 2:**
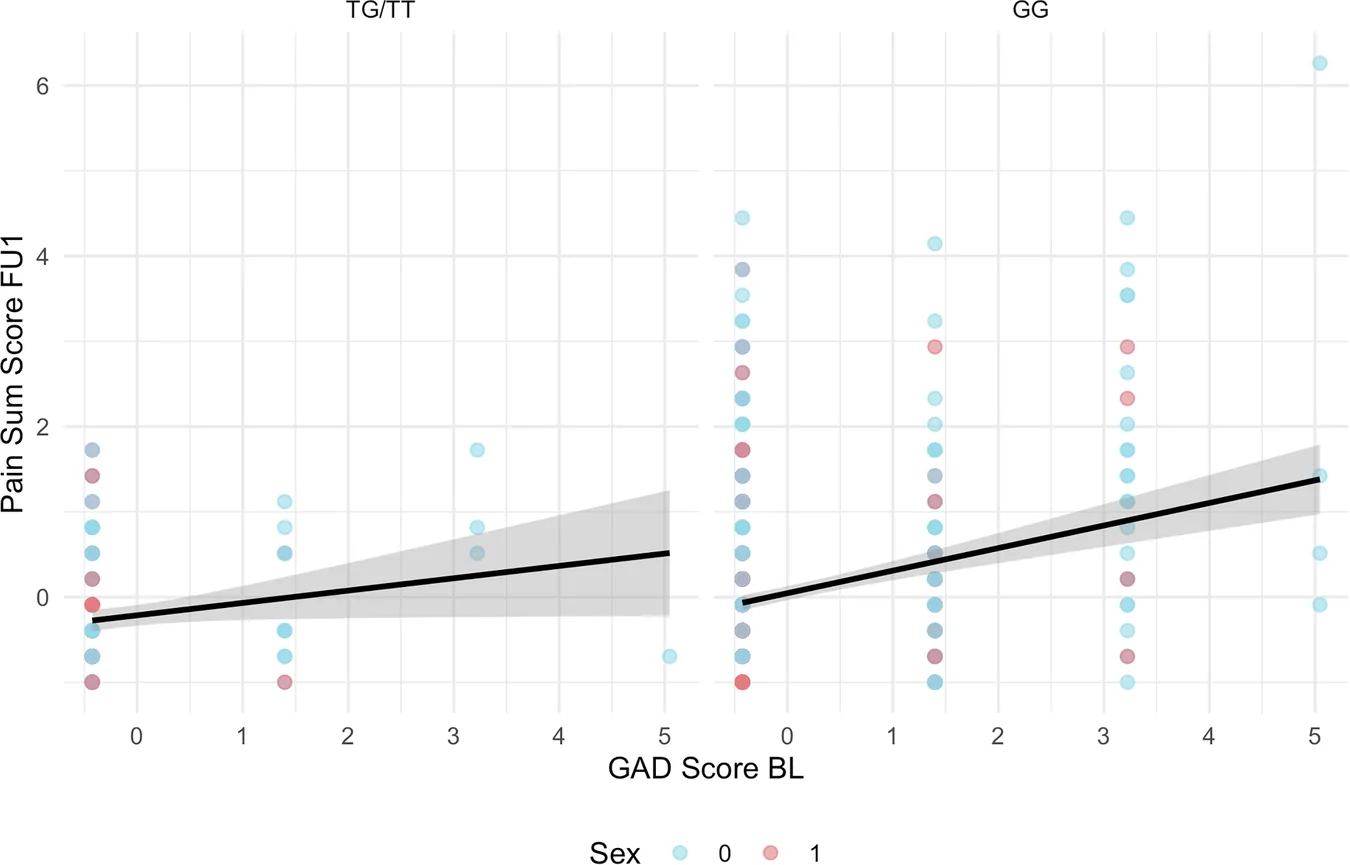
Association between anxiety at age 14 and pain at age 16 differs across rs6721961 *NRF2* variants. Within carriers of GG of rs6721961, pain complaint magnitudes were significantly predicted by anxiety [*β* = 0.238 (95% CI: 0.156, 0.320); *p* = 1.87e-08], with the explained variance = 7.6% (*p* = 1.17e-08). In contrast, no significant association was found in TG/TT carriers (*p* > 0.05). All statistical tests were two-sided. GAD generalized anxiety disorder, BL baseline, FU1 follow-up 1.

These findings suggest potential differences in the relationship between early anxiety and later pain outcomes across rs6721961 genotypes. The association was significant among adolescents with the GG genotype of rs6721961, but not among TG/TT carriers (Fig. 2).

As rs35652124 showed no main effects or differences across genotype groups, it was excluded from further stratified analyses.

## Discussion

In this longitudinal study, we replicated previous findings showing that symptoms of anxiety and depression in mid-adolescence are associated with greater pain complaints two years later. This observation is consistent with earlier population-based studies and further supports the established link between internalising symptoms and later pain outcomes during adolescence^31,32^. While this prospective relationship is increasingly well documented, considerably less is known about the biological factors that may explain why some adolescents with elevated internalising symptoms develop pain, whereas others do not. The principal novelty of the present study, to the best of our knowledge, is that anxiety symptoms and later pain association were linked with genetic variation in NRF2, a transcription factor involved in oxidative stress regulation and immune signaling. Specifically, carriers of the rs6721961 GG genotype exhibited higher anxiety levels and more pain complaints, and the association between anxiety and pain was significant in this genotype group. In contrast, no significant association between anxiety and later pain outcomes was found in carriers of the TG/TT genotypes. These findings suggest that NRF2-related pathways may be potentially associated with individual differences in vulnerability to pain among adolescents experiencing internalising symptoms. This appears consistent with preclinical work identifying NRF2 as a key regulator of susceptibility to affective symptoms and pain-related behaviours in animal models^16,17,33^. Our results also align with evidence indicating that the TG/TT variants of rs6721961 are associated with higher *NRF2* expression^18^, and therefore potentially a greater capacity to regulate neuroinflammatory and oxidative stress responses.

We observed sex differences, with females reporting higher anxiety, depression, and pain - consistent with existing literature^34,35^. However, sex did not moderate the anxiety-pain association or differ across genotype distributions, suggesting that the observed genetic effects are not confounded by sex.

Together, these findings point to the *NRF2* rs6721961 polymorphism as a potential genetic factor that is related to the relationship between early anxiety and later pain. While exploratory, these results contribute to emerging efforts to identify genetically based susceptibility and resilience factors for pain and affective comorbidity.

Several limitations should be acknowledged. First, the absence of baseline pain assessment within the IMAGEN dataset at age 14 restricted our ability to determine the extent to which the observed affect-pain association is independent of pre-existing pain symptoms. This should be investigated in future studies. Second, while our sample was large and longitudinal, genotype frequencies limited the statistical power for certain subgroup analyses. Pain and affective symptoms were self-reported, and clinical diagnoses or biomarkers (e.g., cytokine levels) would strengthen the findings. We focused on two *NRF2* SNPs; other polymorphisms or epigenetic factors may also play a role. Additionally, limitations inherent to candidate gene studies, such as multiple testing issues and low reproducibility, should not be overlooked. Finally, adjusting the regression models for other pain-sensitive factors, such as childhood adversity and sleep, may further improve the models.

In addition to including baseline pain assessment, future studies should examine the neurobiological correlates of these genetic effects using neuroimaging to assess structural and functional connectivity in key brain regions, including the anterior cingulate cortex, insula, amygdala, and hippocampus. Integration with inflammatory biomarkers would also clarify the immune signaling pathways involved.

In conclusion, the present findings suggest that the *NRF2* gene variant rs6721961 is associated with the relationship between early internalising symptoms and later pain during adolescence. Although these findings should not be interpreted as evidence that rs6721961 significantly moderates the association, they highlight the importance of considering genetic heterogeneity when examining complex relationships between psychological and pain-related outcomes and may provide a basis for future research into underlying mechanisms. In addition, while the explained variance is moderate (7.6%), these results align with growing evidence implicating immune and oxidative stress pathways in both emotional and somatic health outcomes. They also support further investigation of NRF2-related genetic variation as a potential contributor to pain vulnerability and as a candidate pathway for understanding early risk trajectories among youth experiencing internalizing symptoms.

## Supplementary information

Transparent Peer Review file

Supplemental Material File

Description of Additional Supplementary Files

Supplementary Data 1

## Data Availability

The present study used data from the IMAGEN consortium; these data are not publicly available. Access to the IMAGEN dataset requires submission of a formal project proposal, which is reviewed and approved by the IMAGEN Project Executive Committee. Further information about the data access and application process is available at https://www.imagen-project.org/dataaccess. Access to and secondary use of the data are subject to the requirements of the relevant local ethics committees and the applicable IMAGEN consortium agreements. The source data for Fig. 1A, B are in Tables 5 and 3, respectively. Meanwhile, the source data for Fig. 2 are in Supplementary Data 1.
