# Supplementary material for: Immune-related gene expression links anxiety, depression, and pain outcomes in adolescence
Source: Commun Med (Lond). 2026 Oct 16;6:539. doi: 10.1038/s43856-026-01960-5 (PMC13675203; doi:10.1038/s43856-026-01960-5)
Supplement: Supplementary file 1 [file 43856_2026_1960_MOESM1_ESM.pdf]

nature portfolio

Peer Review File

Immune-related gene expression links anxiety, depression, and pain outcomes in adolescence

Corresponding Author: Professor Frauke Nees

This file contains all reviewer reports in order by version, followed by all author rebuttals in order by version.

Version 0:

Reviewer comments:

Reviewer #1

(Remarks to the Author)
This manuscript describes a study of 724 adolescents, asking whether there was an association between anxiety/depression symptoms at baseline and a sum of pain complaints at follow up ~2 years later, and whether this relationship differed between SNP genotypes. SNPs were in gene NRF2, a gene with a priori evidence for pain involvement in animal and other studies. The manuscript is for the most part well-written and the results are interesting. I have some concerns

general:
raw p values should be reported throughout rather than just $p < 0.01$, etc.

results:

there is no descriptive table of all possible pain complaints even though pain types resembling neuropathic symptoms are mentioned.

it is also not fully clear how the p values in the second paragraph of 2.2.1 are obtained - Mann-Whitney U tests are non-parametric tests to determine if group values are higher in 1 group vs another - are the authors carrying out repeated Mann-Whitney U tests and separating the total N of pain complains into these categories (i.e., test number 1, does TG/TT have significantly more neuropathic pain complaints than GG, test 2, does TG/TT have significantly more joint pain type complaints than GG, and so on) - if so multiple test correction should be at least discussed. a binomial test of enrichment of particular pain types may also be more appropriate

minor: a table of regression output may be easier for a reader than having all results in-text

limitations:
these should include more discussion of how this is in essence a candidate gene study. while the gene has significant animal study and other suggestive evidence related to its involvement in pain, the caveats of this type of study should be briefly addressed. this is another reason why reporting raw p values is desirable e.g., $p = 0.008$ is not particularly convincing in this scenario, but $p = 5e-6$ is much more so, $p < 0.001$ could be either

methods/approach:
while the authors cite evidence as to why NRF2 gene is chosen, there is no mention of what motivates selection of these two particular SNPs within NRF2, and considering they are 36bp (?) apart, there should be some discussion of how independent they are

I am also curious as to why the authors split the sample by genotype, rather than retaining as much power as possible by regression with genotype (0/1/2 complement of G at rs6721961, for example) and using an interaction term to test whether anxiety-pain or depression-pain relationships are genotype-dependent.

The significant finding for GG rs6721961 could really just be the significantly larger sample size for that genotype subgroup - if the GG subgroup is randomly downsampled to N = 135, does the significant finding remain?

I also think this manuscript's findings would be significantly strengthened by including a negative control (variants in a gene without any strong prior evidence for pain involvement), or by applying permutation to get an empirical test statistic distribution for this small sample size.

Reviewer #2

(Remarks to the Author)
In this analyses of a study of 700+ adolescents that is longitudinal, symptoms of anxiety/depression predicted pain complaints at ages 16-17. The authors also posit that such effects were stronger in NRF2 GG vs TG/TT genotype carriers, though effect sizes for different groups are not present and neither are moderation analyses conducted. These findings may be meaningful but further analyses are needed, given that baseline pain symptoms are controlled/assessed. This may be a major confound, e.g. if GAD at BL and pain at BL are highly correlated and pain at BL and pain at FU1 are highly correlated, then it may be appear as though anxiety/depression is linked to prospective pain symptoms. Moreover, formal moderation are needed, as the lack of findings in the TG/TT genotype groups may be because sample sizes/variance limited statistical significance. Lastly, the choice of genes and incorporation of whether analyses were pre-registered is needed. However, the study size, longitudinal design, and diversity of parameters examined warrant re-submission with major revisions to review these concerns.

Methods section
The study size is a strength

It should be made clear if the present hypotheses were pre-registered as the choices of genes in NRF2 is somewhat vague, given that wide range of genes may affect both pain and depression/anxiety is quite large, and could draw concerns from readers about HARKing. For instance, NFKB1, JUN, SOD1/2 and FOS genes are more well-studied. However, these concerns could be tempered if the findings are framed as exploratory.

The lack of statistical adjustment for baseline pain is a major concern as this may fully confound findings.

Results

The results section is clearly described.

The findings appear potentially clinically meaningful, dependent on methological approaches.

Discussion
In the discussion, the links between anxiety, ACC, and LTP should be framed as speculative since the present study did not test this.

The explanation for the variance (~7%) is fully meaningful, such associations are expected to be small to moderate given the large range of factors that might affect pain symptoms.

Reviewer #3

(Remarks to the Author)
Thank you for the opportunity to review this manuscript. The authors use IMAGEN to test whether anxiety/depression at age 14-15 predict pain at 16-17, and whether two NRF2 SNPs moderate this. The question is worthwhile, and the role of the immune system in the etiology of chronic pain is topical, but in my opinion several issues substantially weaken the main claims.

Major comments
1. There is no baseline pain measure. The models (Eq. 1a/1b) do not adjust for pain at age 14, so reverse causation cannot be ruled out: adolescents with pre-existing pain may simply have higher baseline anxiety/depression. Either adjust for baseline pain or acknowledge this as a fundamental limitation and temper the causal language throughout.
2. Directionality and confounding. The framing assumes affective symptoms drive pain via inflammation, but inflammation could equally be a common cause of both. Models adjust only for sex. Other factors, such as childhood adversity, SES, and sleeo, are not considered.
3. A priori SNP selection. It is not clear whether NRF2 and these two SNPs were chosen in advance. With ~583,000 markers available, I would expect a pre-registered plan or clear a priori rationale.
4. Moderation vs. mediation, and stratified vs. interaction. The authors use these terms interchangeably, but a SNP cannot mediate an effect of anxiety, as it precedes anxiety. More importantly, what is described as moderation is in fact a stratified analysis comparing p-values across genotypes. This is not a valid test of moderation. If the authors wish to assess effect modification by genotype, a single model with an anxiety-by-genotype interaction term should be fitted, with the interaction coefficient reported.
5. Genetic model. With only 5 TT carriers, collapsing TG+TT assumes a dominant model without justification, and

dominant/additive/recessive models cannot really be distinguished here. Please justify the coding and provide sensitivity analyses.
6. Population stratification. IMAGEN is multi-site (Germany, France, UK, and Ireland). Principal components and study site should be included as covariates in the main models, not only in upstream QC.
7. Multiple testing. Bonferroni correction is claimed but the test family is unspecified, and reported p-values do not appear corrected? Both uncorrected and corrected p-values should be shown.
8. The ACC/LTP/cytokine narrative in the Discussion is attached to a study that measured none of these. Given that IMAGEN has neuroimaging, the paper would be significantly stronger if imaging data were used.
9. Attrition and selection bias. IMAGEN's full baseline cohort is ~2,000. However, the authors analyze 724 with complete baseline + follow-up + genotype data. No comparison of included vs. excluded participants on key variables (baseline anxiety, depression, SES, site). Differential attrition by affective symptoms is plausible and would introduce bias into the exposure-outcome association in this study.
10. Finally, that anxiety/depression precede pain in adolescence is already reasonably well established (indeed, the authors cite existing evidence of this). Instead, the novel contribution in this study is the NRF2 finding, which should be framed as such. However, this finding itself rests on a stratified analysis (with no test for effect modification) in a rather modest sample size (N=724; only 135 TG/TT), and which has rather important methodological issues.

Other minor comments
Bolding for emphasis in Results is distracting; italicisation of NRF2 (gene vs. protein) is inconsistent; the CSI pain subscale items and internal consistency should be reported; DAWBA output is ordinal and should not be treated as continuous without acknowledgement.

In summary, the two most important issues are the absence of a baseline pain measure and the use of stratified rather than interaction analysis. Together these mean the headline claim (that immune dysfunction explains the association between anxiety and depression and later pain) are not properly tested.

Version 1:

Reviewer comments:

Reviewer #1

(Remarks to the Author)
I thank the authors for their thorough rebuttal letter. My concerns have been addressed

Reviewer #2

(Remarks to the Author)
The authors have satisfactorily addressed my concerns.

Reviewer #3

(Remarks to the Author)
Thank you for providing this updated manuscript, which I enjoyed reviewing. I thank the authors for the additional analyses which are welcome (negative-control SNP, permutation test, attrition comparison, site adjustment, and reporting of raw p-values). However, one of my principle concerns remains substantively unaddressed, which I feel is important as it is central to the message of this manuscript.

Stratified analysis vs. test of effect modification: My original comment was that a moderation claim cannot be supported by comparing a significant association in one genotype group against a non-significant one in another (i.e. a stratified analysis as reported here). Although the revision relabels terminology to some extent, it retains the moderation framing while still resting entirely on stratified models (see Table 4, Models 3 and 4), and no interaction term has been fitted.

This is exemplified in the Results section under heading "Moderation of the Anxiety-Pain Association by NRF2 (rs6721961)", which illustrates the issue. The authors describe "stratified regression analyses" and then conclude that the relationship is "genotype-dependent" and "absent in TG/TT carriers". This states a moderation claim which is never tested. The "absent in TG/TT" characterisation is also not supported by the authors' own Table 4, where the TG/TT slope (beta=0.133 [-0.013, 0.278]) is in the same direction, of similar magnitude, and overlaps substantially with the GG slope (beta=0.238 [0.156, 0.320]). This pattern could also be consistent with the smaller subgroup (N=135) being underpowered rather than with an absent effect, and without formally testing an anxiety×genotype interaction term we cannot know. Moreover, the closing statement that rs35652124 was excluded because it "showed no... gene-phenotype interactions" implies an interaction test for this SNP that does not appear for rs6721961.

Relatedly, the Abstract states that "gene-by-phenotype interactions" were tested, however no interaction tests appear in any model.

(I would also note that the additional permutation and down-sampling analyses, while welcome, test genotype differences in pain level and the robustness of the GG slope respectively. Neither tests whether the slope, which is of primary interest to

their hypothesis, differs by genotype.)

The stated objective of the paper is to investigate "whether two common NRF2 polymorphisms (rs6721961 and rs35652124) moderate the association between anxiety and depression symptoms at age 14-15 and pain complaints two years later at age 16–17."

Given the focus on moderation, I would ask the authors to fit and report a formal anxiety×genotype interaction coefficient, to formally test moderation by genotype.

Alternatively, they should remove all effect-modification language ("moderated," "genotype-dependent," "absent in TG/TT," "resilient") and present the result strictly as a stratified descriptive analysis.

Finally, regarding reverse causation: I accept the authors explanation that baseline pain was not assessed in IMAGEN and therefore cannot be adjusted for. My remaining concern is therefore one of framing rather than analysis as the causal framing has not been brought into line with this limitation. Phrases such as "affect-induced pain", "confer greater vulnerability", "developmental trajectory from early anxiety to later pain", and "target for early intervention" remain throughout. Given that pre-existing pain could plausibly elevate baseline anxiety and track forward to follow-up pain, I would ask that this limitation be foregrounded as a primary limitation and that causal language be removed rather than balanced against a later caveat in the discussion.

Version 2:

Reviewer comments:

Reviewer #3

(Remarks to the Author)
Thank you for providing this revised manuscript and for your careful response to my comments on the previous draft. I am satisfied that my principal concerns have now been substantially addressed, particularly through reframing the genotype analyses as stratified rather than as evidence of formal moderation, and by appropriately foregrounding the absence of baseline pain as a major limitation.

I would, however, suggest one final tightening of the language around the genotype findings: given that no formal anxiety-by-genotype interaction has been demonstrated, statements that the association appears “specific” to GG carriers or that the relationship differs between genotype groups should remain explicitly descriptive.

The absence of baseline pain also substantially limits the extent to which the temporal association can be interpreted as anxiety preceding the development of pain. Subject to these relatively minor points, I think the manuscript is considerably improved.

Open Access This Peer Review File is licensed under a Creative Commons Attribution 4.0 International License, which

permits use, sharing, adaptation, distribution and reproduction in any medium or format, as long as you give appropriate credit to the original author(s) and the source, provide a link to the Creative Commons license, and indicate if changes were made.
In cases where reviewers are anonymous, credit should be given to 'Anonymous Referee' and the source.
The images or other third party material in this Peer Review File are included in the article's Creative Commons license, unless indicated otherwise in a credit line to the material. If material is not included in the article's Creative Commons license and your intended use is not permitted by statutory regulation or exceeds the permitted use, you will need to obtain permission directly from the copyright holder.
To view a copy of this license, visit https://creativecommons.org/licenses/by/4.0/

Referee #1: Chronic pain; Genetics; Psychiatry

Referee #2: Psychiatry; Inflammation

Referee #3: Epidemiology; Pain; Neuroinflammation

Reviewers' comments:

Reviewer #1 (Remarks to the Author):

Reviewer comment:

This manuscript describes a study of 724 adolescents, asking whether there was an association between anxiety/depression symptoms at baseline and a sum of pain complaints at follow up ~2 years later, and whether this relationship differed between SNP genotypes. SNPs were in gene NRF2, a gene with a priori evidence for pain involvement in animal and other studies. The manuscript is for the most part well-written and the results are interesting. I have some concerns

Reply: Thank you very much for the constructive comments, which greatly helped to improve our manuscript. Please find our detailed replies to each individual comment below.

Reviewer comment:

general: raw p values should be reported throughout rather than just $p < 0.01$, etc.

Reply: Thank you for your feedback. We have now provided raw p-values and adjusted p-values accordingly.

Reviewer comment:

results: there is no descriptive table of all possible pain complaints even though pain types resembling neuropathic symptoms are mentioned.

Reply: We have now included the descriptive table of all pain/weakness items. Please refer to Table 1 and Table 2.

Reviewer comment:

it is also not fully clear how the p values in the second paragraph of 2.2.1 are obtained - Mann-Whitney U tests are non-parametric tests to determine if group values are higher in 1 group vs another – are the authors carrying out repeated Mann-Whitney U tests and separating the total N of pain complains into these categories (i.e., test number 1, does TG/TT have significantly more neuropathic pain complaints than GG, test 2, does TG/TT have significantly more joint pain type complaints than GG, and so on) – if so multiple test correction should be at least discussed. A binomial test of enrichment of particular pain types may also be more appropriate.

Reply:

We thank the reviewer for this comment. We clarify that, for each pain item, the participant response format is a 5-point scale ranging from "*not at all*" (0) to "*a whole lot*" (4). Please refer to **Method 4.3** for our description:

"The CSI consists of 35 items, including the somatic symptoms defined by DSM-III-R (American Psychiatric Association, 1987), the Hopkins Symptom Checklist (HSCL)[30], and additional symptoms common in functional gastrointestinal disorders. The response format is a 5-point scale ranging from "not at all" (0) to "a whole lot" (4), which results in a range of sum scores from 0 to 140, and the standard period for symptom reports is 2 weeks. Items that are related to pain and motor weakness symptoms were selected based on ref.[31]."

Differences in each pain item between genotype groups were then assessed using separate Mann–Whitney U tests.

We agree that this involves multiple comparisons and have applied the Benjamini–Hochberg procedure to adjust p-values. This has been clarified in the revised Methods section. Please also refer to Table 1 for the outcome.

Regarding this, we also toned down and clarified our report on this part.

"Notably, this difference appeared to be pronounced for pain types resembling neuropathic symptoms, including pain in arms/legs (p = 3.734e-03), numbness/tingling (p = 3.654e-02), and, to a lesser extent, joint pain (p = 1.899e-02). However, only pain in arms/legs survived multiple testing corrections (Table 1)."

Reviewer comment:

minor: a table of regression output may be easier for a reader than having all results in-text

Reply:

Thank you for the helpful feedback. We have now included a table of regression output. Please refer to Table 4.

Reviewer comment:

limitations: these should include more discussion of how this is in essence a candidate gene study. while the gene has significant animal study and other suggestive evidence related to its involvement in pain, the caveats of this type of study should be briefly addressed. this is another reason why reporting raw p values is desirable e.g., $p = 0.008$ is not particularly convincing in this scenario, but $p = 5e\text{-}6$ is much more so, $p < 0.001$ could be either

Reply:

We appreciate this comment from the reviewer and have added the raw p value accordingly in the manuscript.

In addition, we also updated the limitation part in the discussion following the reviewer's advice:

"Several limitations should be acknowledged. First, while our sample was large and longitudinal, genotype frequencies limited the statistical power for certain subgroup analyses. Next, the absence of baseline pain assessment within the IMAGEN dataset at age 14 restricted our ability to determine the extent to which the observed affect-pain association is independent of pre-existing pain symptoms. Pain and affective symptoms were self-reported, and clinical diagnoses or biomarkers (e.g., cytokine levels) would strengthen causal inference. We focused on two NRF2 SNPs; other polymorphisms or epigenetic factors may also play a role. In addition, as a candidate gene study, limitations inherent to this approach, including multiple testing issues and low reproducibility, should not be overlooked. Adjusting the regression models for other pain-sensitive factors, such as childhood adversity and sleep, in addition to sex and sample collection sites, could also improve the models."

Reviewer comment:

methods/approach: while the authors cite evidence as to why NRF2 gene is chosen, there is no mention of what motivates selection of these two particular SNPs within NRF2, and considering they are 36bp (?) apart, there should be some discussion of how independent they are

Reply:

Thank you for this comment. We have now revised the manuscript accordingly to provide more details on the rationales of choosing NRF2 and its two SNPs. Please find the text below.

"A candidate molecular regulator in this context is nuclear factor erythroid 2–related factor 2 (NRF2) – a transcription factor that orchestrates antioxidant and anti-inflammatory responses[14]. Activation of NRF2 has been shown to mitigate both pain behaviours and affective symptoms in animal models[15,16]. In addition, our recent work suggests that NRF2 may contribute to individual susceptibility and resilience to chronic pain and its affective comorbidity [17]. At the genetic level, promoter-region polymorphisms such as rs6721961 and rs35652124, which have been linked to differences in predicted regulatory motifs, have been associated with altered NRF2 expression and transcriptional activity, potentially influencing inflammatory and oxidative stress responses[18–21]. These variants therefore represent plausible candidates for moderating the link between internalising symptoms and later pain outcomes. Despite accumulating evidence for prospective associations between internalising symptoms and pain, and growing evidence implicating NRF2 in both affective and pain-related processes, it remains unknown whether genetic variation in NRF2 contributes to individual differences in the strength of this association during adolescence."

Reviewer comment:

I am also curious as to why the authors split the sample by genotype, rather than retaining as much power as possible by regression with genotype (0/1/2 complement of G at rs6721961, for example) and using an interaction term to test whether anxiety-pain or depression-pain relationships are genotype-dependent.

Reply:

Thank you for the suggestion. We totally understand the point raised by the reviewer. However, for our objective, which is to investigate whether *NRF2* polymorphisms influence the prospective association between anxiety and depression symptoms at age 14-15 and pain complaints two years later, the group analyses are the main outcome. We have now adjusted the terminology throughout the manuscript, including in the Abstract, Introduction, Results, and Discussion sections.

Reviewer comment:

The significant finding for GG rs6721961 could really just be the significantly larger sample size for that genotype subgroup - if the GG subgroup is randomly downsampled to N = 135, does the significant finding remain?

Reply:

Thank you for your feedback. The significant finding remains when the GG subgroup is randomly downsampled. Please find the details in Table S1.

Reviewer comment:

I also think this manuscript's findings would be significantly strengthened by including a negative control (variants in a gene without any strong prior evidence for pain involvement), or by applying permutation to get an empirical test statistic distribution for this small sample size.

Reply:

Thank you very much for your suggestion. We have now included a negative control and also applied permutation.

A permutation test (10000 permutations) showed that the observed difference in mean pain sum score between rs6721961 genotypes was unlikely under the null distribution (empirical p = 0.0022).

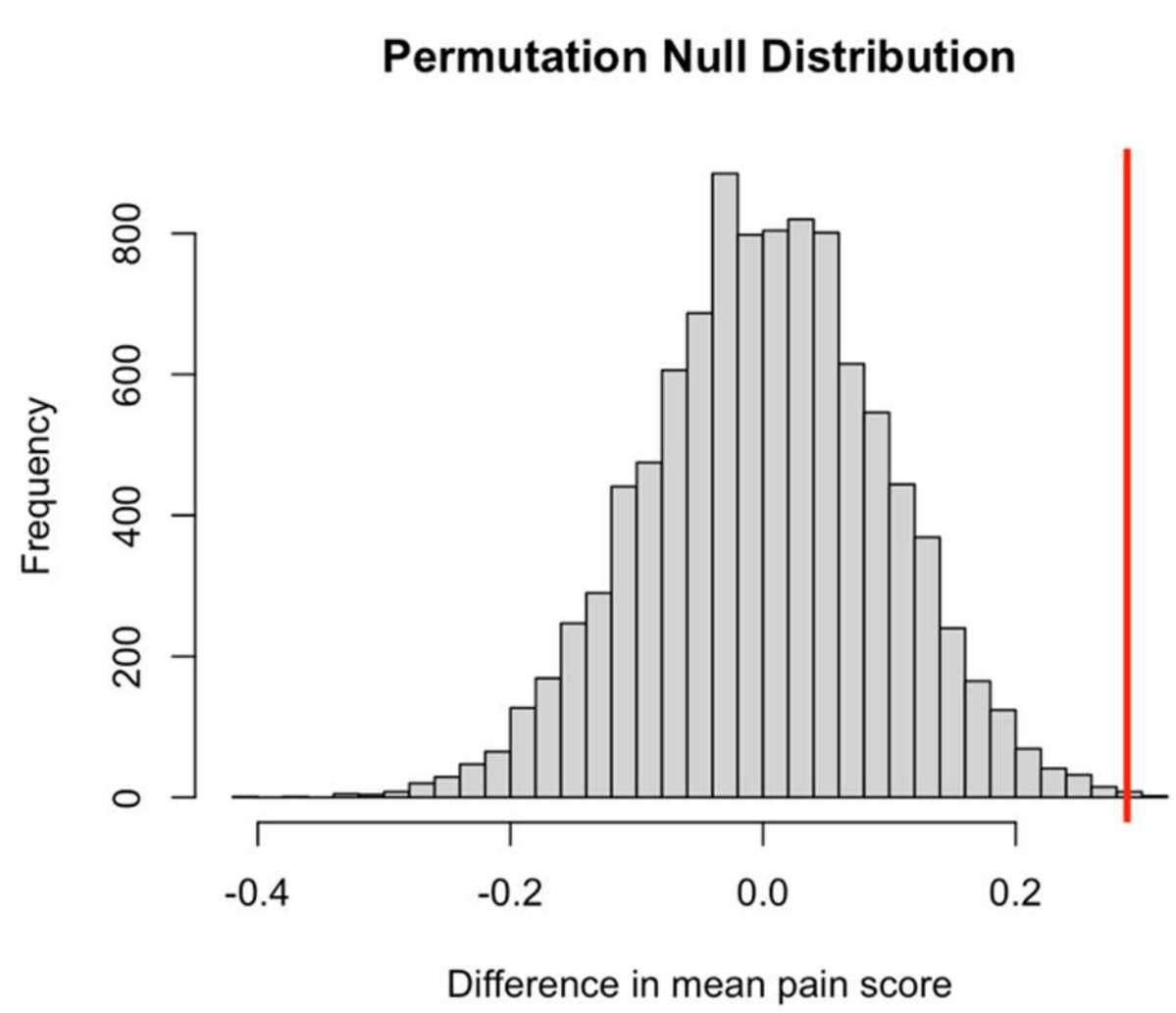

*The red line represents the observed difference

We have adjusted the manuscript accordingly in the Methods section 4.4.

"For this study, we report data on 589 GG genotype carriers of rs6721961, 130 TG, and 5 TT genotype carriers. Due to a limited number of the TT allele, TG and TT will be merged (N=135). Meanwhile, the SNP rs35652124 consists of 337 TT, 312 CT, and 75 CC. Please refer to Table 6 for more details on the SNPs. The present hypotheses were not pre-registered. However, the selection of NRF2 and its SNPs was guided by a priori biological considerations and based on the established role of NRF2 in oxidative stress and immune response regulation implicated in both pain and affective symptoms.
To access the robustness and specificity of the outcome, analyses were also conducted using NRF2 rs2886161 as a negative control, supplemented with permutation testing. Compared to the two main SNPs, the negative control is located in a different region (Intron 1) in the NRF2 gene and has few prior studies linking it with pain and comorbidities [34]."

And the result part:

"A permutation test (10000 permutations) showed that the observed difference in mean pain sum score between rs6721961 genotypes was unlikely under the null distribution (empirical p = 0.0022). Meanwhile, the total pain scores were not significantly different between alleles of the negative control rs2886161 (Please see the supplemental material for details)."

Reviewer #2 (Remarks to the Author):

Reviewer comment:

In this analyses of a study of 700+ adolescents that is longitudinal, symptoms of anxiety/depression predicted pain complaints at ages 16-17. The authors also posit that such effects were stronger in NRF2 GG vs TG/TT genotype carriers, though effect sizes for different groups are not present and neither are moderation analyses conducted.

These findings may be meaningful but further analyses are needed, given that baseline pain symptoms are controlled/assessed. This may be a major confound, e.g. if GAD at BL and pain at BL are highly correlated and pain at BL and pain at FU1 are highly correlated, then it may be appear as though anxiety/depression is linked to prospective pain symptoms. Moreover, formal moderation are needed, as the lack of findings in the TG/TT genotype groups may be because sample sizes/variance limited statistical significance. Lastly, the choice of genes and incorporation of whether analyses were pre-registered is needed. However, the study size, longitudinal design, and diversity of parameters examined warrant re-submission with major revisions to review these concerns.

Reply:

Thank you for all your feedback, which greatly helped us to improve this manuscript. We have addressed each point below following your advice.

Regarding the genotype subgroup analysis, in the revised submission, we included additional analyses and demonstrated that when the GG subgroup is randomly downsampled to 135 (matching the TG/TT subgroup), the significant finding remains. Please find Table S1 for more details.

In terms of the analyses, we would also like to clarify that the primary objective of this study was to investigate whether *NRF2* polymorphisms influence the prospective association between anxiety and depressive symptoms at age 14-15 years and pain complaints two years later. Accordingly, the genotype-stratified analyses constitute the primary outcome of interest. To better reflect this focus, the terminology has been revised throughout the manuscript, including in the Abstract, Introduction, Results, and Discussion sections.

Reviewer comment:

Methods section

The study size is a strength. It should be made clear if the present hypotheses were pre-registered as the choices of genes in NRF2 is somewhat vague, given that wide range of genes may affect both pain and depression/anxiety is quite large, and could draw concerns from readers about HARKing. For instance, NFKB1, JUN, SOD1/2 and FOS genes are more well-studied. However, these concerns could be tempered if the findings are framed as exploratory.

Reply:

We thank the reviewer for this important comment. The present hypotheses were not pre-registered. However, the selection of NRF2 was based on a priori biological considerations, and this has now been explicitly stated in the manuscript.

Specifically, we have improved the clarification on the choice of NRF2 and its SNPs.

Apart from emerging evidence linking *NRF2* function and pathophysiology of (chronic) pain as well as affective symptoms, our recent work demonstrated that this transcriptional factor appears to contribute to susceptibility and resilience to chronic pain and its affective comorbidity.

Le, A.C.T., Fiuza-Fernandes, J., Silva, J.M., Sampaio, M.T., Texeira-Castro, A., Duarte-Silva, S., Leite-Almeida, H., 2025. Pretreatment with dimethyl fumarate prevents chronic pain and its comorbidities via Nrf2 pathway in a rat model of neuropathic pain. Brain. Behav. Immun. 128, 725–736. https://doi.org/10.1016/j.bbi.2025.05.003

In addition, to further address concerns regarding spurious associations, we included analyses of a negative-control SNP (rs2886161) and conducted permutation testing.

We made revisions accordingly in the manuscript:
In the introduction:

"*A candidate molecular regulator in this context is nuclear factor erythroid 2-related factor 2 (NRF2) – a transcription factor that orchestrates antioxidant and anti-inflammatory responses[14]. Activation of NRF2 has been shown to mitigate both pain behaviours and affective symptoms in animal models[15,16]. In addition, our recent work suggests that NRF2 may contribute to individual susceptibility and resilience to chronic pain and its affective comorbidity [17]. At the genetic level, promoter-region polymorphisms such as rs6721961 and rs35652124, which have been linked to differences in predicted regulatory motifs, have been associated with altered NRF2 expression and transcriptional activity, potentially influencing inflammatory and oxidative stress responses[18–21]. These variants therefore represent plausible candidates for moderating the link between internalising symptoms and later pain outcomes. Despite accumulating evidence for prospective associations between internalising symptoms and pain, and growing evidence implicating NRF2 in both affective and pain-related processes, it remains unknown whether genetic variation in NRF2 contributes to individual differences in the strength of this association during adolescence.*"

In the Methods section 4.4:

"*For this study, we report data on 589 GG genotype carriers of rs6721961, 130 TG, and 5 TT genotype carriers. Due to a limited number of the TT allele, TG and TT will be merged (N=135). Meanwhile, the SNP rs35652124 consists of 337 TT, 312 CT, and 75 CC. Please refer to Table 6 for more details on the SNPs. The present hypotheses were not pre-registered. However, the selection of NRF2 and its SNPs was guided by a priori biological considerations and based on the established role of NRF2 in oxidative stress and immune response regulation implicated in both pain and affective symptoms.*
To access the robustness and specificity of the outcome, analyses were also conducted using NRF2 rs2886161 as a negative control, supplemented with permutation testing. Compared to the two main SNPs, the negative control is located in a different region (Intron 1) in the NRF2 gene and has few prior studies linking it with pain and comorbidities [34]."

Furthermore, while emphasizing the importance of the findings on novel factors contributing to pain development, we have acknowledged that the findings are considered exploratory:

"Collectively, our findings point to NRF2 rs6721961 polymorphism as a potential novel moderator in the developmental trajectory from early anxiety to later pain, and to NRF2-mediated immune modulation as a plausible biological mechanism underlying this relationship. While exploratory, these results contribute to emerging efforts to identify genetically based susceptibility and resilience factors for pain and affective comorbidity"

Reviewer comment:

The lack of statistical adjustment for baseline pain is a major concern as this may fully confound findings.

Reply:

Thank you for this important comment. We agree that adjustment for baseline pain would strengthen the interpretation of the prospective associations. However, pain symptoms were not assessed at baseline (age 14-15) in the IMAGEN cohort and therefore could not be included in the present analyses.

Importantly, the primary objective of this study was to investigate whether anxiety and depression symptoms at age 14-15 predict pain complaints at age 16-17, rather than to examine changes in pain relative to an earlier assessment. Thus, pain complaints at age 16-17 constituted the predefined outcome of interest. While baseline pain information would have allowed us to determine the extent to which these associations are independent of pre-existing pain symptoms, its absence does not preclude the evaluation of prospective associations between adolescent internalizing symptoms and subsequent pain outcomes.

We also note that establishing a single "baseline" for pain during adolescence is inherently challenging, given that pain symptoms can fluctuate substantially across developmental stages and may not be adequately represented by a single assessment point. Our focus was therefore on pain outcomes at age 16-17 as a clinically relevant developmental period.

Nevertheless, the lack of baseline pain measurements is an important limitation. Specifically, we cannot determine whether the observed associations reflect the emergence of new pain complaints or the persistence of pre-existing symptoms.

Consequently, the findings should be interpreted as evidence of prospective associations rather than definitive evidence of causal effects.

We have tempered the causal language throughout the manuscript and now explicitly acknowledge this limitation. Future studies including repeated pain assessments across adolescence will be important to disentangle the development and persistence of pain symptoms over time.

In addition, in the limitation part of the discussion, we have now acknowledged this:

"Several limitations should be acknowledged. First, while our sample was large and longitudinal, genotype frequencies limited the statistical power for certain subgroup analyses. Next, the absence of baseline pain assessment within the IMAGEN dataset at age 14 restricted our ability to determine the extent to which the observed affect-pain association is independent of pre-existing pain symptoms. Pain and affective symptoms were self-reported, and clinical diagnoses or biomarkers (e.g., cytokine levels) would strengthen causal inference. We focused on two NRF2 SNPs; other polymorphisms or epigenetic factors may also play a role. In addition, as a candidate gene study, limitations inherent to this approach, including multiple testing issues and low reproducibility, should not be overlooked. Adjusting the regression models for other pain-sensitive factors, such as childhood adversity and sleep, in addition to sex and sample collection sites, could also improve the models."

As well as provide suggestions for future studies:

"*In addition, studies that include repeated pain assessments across adolescence will be important for disentangling the development and persistence of pain symptoms over time.*"

Reviewer comment:

Results

The results section is clearly described.

The findings appear potentially clinically meaningful, dependent on methodological approaches.

Discussion

In the discussion, the links between anxiety, ACC, and LTP should be framed as speculative since the present study did not test this.

The explanation for the variance (~7%) is fully meaningful, such associations are expected to be small to moderate given the large range of factors that might affect pain symptoms.

Reply:

We appreciate the reviewer's positive comments and have now adjusted the manuscript accordingly.

Reviewer #3 (Remarks to the Author):

Reviewer comment:

Thank you for the opportunity to review this manuscript. The authors use IMAGEN to test whether anxiety/depression at age 14-15 predict pain at 16-17, and whether two NRF2 SNPs moderate this. The question is worthwhile, and the role of the immune system in the etiology of chronic pain is topical, but in my opinion several issues substantially weaken the main claims.

Reply:

Thank you for your comments, which truly helped us to improve this manuscript. We are also grateful to have you as the reviewer.

Reviewer comment:

1. There is no baseline pain measure. The models (Eq. 1a/1b) do not adjust for pain at age 14, so reverse causation cannot be ruled out: adolescents with pre-existing pain may simply have higher baseline anxiety/depression. Either adjust for baseline pain or acknowledge this as a fundamental limitation and temper the causal language throughout.

Reply:

Thank you for this important comment. We agree that adjustment for baseline pain would strengthen the interpretation of the prospective associations. However, pain symptoms were not assessed at baseline (age 14-15) in the IMAGEN cohort and therefore could not be included in the present analyses.

Importantly, the primary objective of this study was to investigate whether anxiety and depression symptoms at age 14-15 predict pain complaints at age 16-17, rather than to examine changes in pain relative to an earlier assessment. Thus, pain complaints at age 16-17 constituted the predefined outcome of interest. While baseline pain information would have allowed us to determine the extent to which these associations are independent of pre-existing pain symptoms, its absence does not preclude the evaluation of prospective associations between adolescent internalizing symptoms and subsequent pain outcomes.

We also note that establishing a single "baseline" for pain during adolescence is inherently challenging, given that pain symptoms can fluctuate substantially across developmental stages and may not be adequately represented by a single assessment point. Our focus was therefore on pain outcomes at age 16-17 as a clinically relevant developmental period.

Nevertheless, the lack of baseline pain measurements is an important limitation. Specifically, we cannot determine whether the observed associations reflect the emergence of new pain complaints or the persistence of pre-existing symptoms.

Consequently, the findings should be interpreted as evidence of prospective associations rather than definitive evidence of causal effects.

We have tempered the causal language throughout the manuscript and now explicitly acknowledge this limitation. Future studies including repeated pain assessments across adolescence will be important to disentangle the development and persistence of pain symptoms over time.

In addition, in the limitation part of the discussion, we have now acknowledged this:

"Several limitations should be acknowledged. First, while our sample was large and longitudinal, genotype frequencies limited the statistical power for certain subgroup analyses. Next, the absence of baseline pain assessment within the IMAGEN dataset at age 14 restricted our ability to determine the extent to which the observed affect-pain association is independent of pre-existing pain symptoms. Pain and affective symptoms were self-reported, and clinical diagnoses or biomarkers (e.g., cytokine levels) would strengthen causal inference. We focused on two NRF2 SNPs; other polymorphisms or epigenetic factors may also play a role. In addition, as a candidate gene study, limitations inherent to this approach, including multiple testing issues and low reproducibility, should not be overlooked. Adjusting the regression models for other pain-sensitive factors, such as childhood adversity and sleep, in addition to sex and sample collection sites, could also improve the models."

As well as provide suggestions for future studies:

"*In addition, studies that include repeated pain assessments across adolescence will be important for disentangling the development and persistence of pain symptoms over time."*

Reviewer comment:

2. Directionality and confounding. The framing assumes affective symptoms drive pain via inflammation, but inflammation could equally be a common cause of both. Models adjust only for sex. Other factors, such as childhood adversity, SES, and sleep, are not considered.

Reply:

Thank you for this feedback. It is true that evidence shows that the immune system can be involved in both the pathophysiology of pain and affective symptoms. In this manuscript, we suggest that among adolescents who experienced internalizing symptoms, those who possess an *NRF2* polymorphism with potentially higher anti-inflammatory and antioxidant activity appeared to have a lower risk of developing future pain. By suggesting the involvement of NRF2 genes, we aim to contribute to emerging efforts to identify genetically based susceptibility and resilience factors for pain and affective comorbidity.

While emphasizing the importance of the findings on novel factors contributing to pain development, we acknowledge in our manuscript that this is an exploratory study.

"*Collectively, our findings point to NRF2 rs6721961 polymorphism as a potential novel moderator in the developmental trajectory from early anxiety to later pain, and to NRF2-mediated immune modulation as a plausible biological mechanism underlying this relationship. While exploratory, these results contribute to emerging efforts to identify genetically based susceptibility and resilience factors for pain and affective comorbidity*."

We have now also added other covariates, such as center sites, to the models, while agreeing that childhood adversity and sleep could also be contributors. Regarding this, we have adjusted the manuscript accordingly and provided the updated outcome in the revised submission.

Reviewer comment:

3. A priori SNP selection. It is not clear whether NRF2 and these two SNPs were chosen in advance. With ~583,000 markers available, I would expect a pre-registered plan or clear a priori rationale.

Reply:

Thank you for this helpful feedback. We totally agree with this point of view.

In the present study, the hypotheses were not pre-registered. However, the selection of NRF2 was based on a priori biological considerations, and this has now been explicitly stated in the manuscript.

Specifically, we have improved the clarification on the choice of NRF2 and its SNPs.

Apart from emerging evidence linking NRF2 function and pathophysiology of (chronic) pain as well as affective symptoms, our recent work demonstrated that this transcriptional factor appears to contribute to susceptibility and resilience to chronic pain and its affective comorbidity.

Le, A.C.T., Fiuza-Fernandes, J., Silva, J.M., Sampaio, M.T., Texeira-Castro, A., Duarte-Silva, S., Leite-Almeida, H., 2025. Pretreatment with dimethyl fumarate prevents chronic pain and its comorbidities via Nrf2 pathway in a rat model of neuropathic pain. Brain. Behav. Immun. 128, 725–736. https://doi.org/10.1016/j.bbi.2025.05.003

In addition, to further address concerns regarding spurious associations, we included analyses of a negative-control SNP (rs2886161) and conducted permutation testing.

We made revisions accordingly in the manuscript:

In the introduction:

"*A candidate molecular regulator in this context is nuclear factor erythroid 2-related factor 2 (NRF2****)*** *– a transcription factor that orchestrates antioxidant and anti-inflammatory responses*[14]. *Activation of NRF2 has been shown to mitigate both pain behaviours and affective symptoms in animal models*[15,16]. *In addition, our recent work suggests that NRF2 may contribute to individual susceptibility and resilience to chronic pain and its affective comorbidity* [17]. *At the genetic level,*

promoter-region polymorphisms such as rs6721961 and rs35652124, which have been linked to differences in predicted regulatory motifs, have been associated with altered NRF2 expression and transcriptional activity, potentially influencing inflammatory and oxidative stress responses[18–21]. *These variants therefore represent plausible candidates for moderating the link between internalising symptoms and later pain outcomes. Despite accumulating evidence for prospective associations between internalising symptoms and pain, and growing evidence implicating NRF2 in both affective and pain-related processes, it remains unknown whether genetic variation in NRF2 contributes to individual differences in the strength of this association during adolescence.*"

In the Methods section 4.4:

"*For this study, we report data on 589 GG genotype carriers of rs6721961, 130 TG, and 5 TT genotype carriers. Due to a limited number of the TT allele, TG and TT will be merged (N=135). Meanwhile, the SNP rs35652124 consists of 337 TT, 312 CT, and 75 CC. Please refer to Table 6 for more details on the SNPs. The present hypotheses were not pre-registered. However, the selection of NRF2 and its SNPs was guided by a priori biological considerations and based on the established role of NRF2 in oxidative stress and immune response regulation implicated in both pain and affective symptoms.*

To access the robustness and specificity of the outcome, analyses were also conducted using NRF2 rs2886161 as a negative control, supplemented with permutation testing. Compared to the two main SNPs, the negative control is located in a different region (Intron 1) in the NRF2 gene and has few prior studies linking it with pain and comorbidities [34]"

Reviewer comment:

4. Moderation vs. mediation, and stratified vs. interaction. The authors use these terms interchangeably, but a SNP cannot mediate an effect of anxiety, as it precedes anxiety. More importantly, what is described as moderation is in fact a stratified analysis comparing p-values across genotypes. This is not a valid test of moderation. If the authors wish to assess effect modification by genotype, a single model with an anxiety-by-genotype interaction term should be fitted, with the interaction coefficient reported.

Reply:

We thank the reviewer for this important comment and apologize for not being precise enough here. We agree that genotype cannot mediate the relationship between anxiety and pain outcomes and have therefore removed mediation-related terminology throughout the manuscript. We haven adjusted the terminology throughout the manuscript.

Regarding the analyses, we totally understand the point raised by the reviewer. However, we would like to clarify that the primary objective of this study was to investigate whether *NRF2* polymorphisms influence the prospective association between anxiety and depressive symptoms at age 14-15 years and pain complaints two years later. Accordingly, the genotype-stratified

analyses constitute the primary outcome of interest. To better reflect this focus, the terminology has been revised throughout the manuscript, including in the Abstract, Introduction, Results, and Discussion sections.

Reviewer comment:

5. Genetic model. With only 5 TT carriers, collapsing TG+TT assumes a dominant model without justification, and dominant/additive/recessive models cannot really be distinguished here. Please justify the coding and provide sensitivity analyses.

Reply:

We thank the reviewer for this important point. We would like to clarify that, in our analyses, SNP was used as a stratification variable rather than a predictor. Because only five participants carried the TT, we combined TG and TT to ensure a sufficient sample size and statistical power, as we did in our previous study.

Nees, F., Becker, S., Millenet, S., Banaschewski, T., Poustka, L., Bokde, A., Bromberg, U., Büchel, C., Conrod, P.J., Desrivières, S., Frouin, V., Gallinat, J., Garavan, H., Heinz, A., Ittermann, B., Martinot, J.L., Papadopoulos Orfanos, Di., Paus, T., Smolka, M.N., Walter, H., Whelan, R., Schumann, G., Flor, H., 2017. Brain substrates of reward processing and the μ-opioid receptor: A pathway into pain? Pain 158, 212–219. https://doi.org/10.1097/J.PAIN.0000000000000720

Reviewer comment:

6. Population stratification. IMAGEN is multi-site (Germany, France, UK, and Ireland). Principal components and study site should be included as covariates in the main models, not only in upstream QC.

Reply:

Thank you very much. We totally agree and have now also added center sites as a covariate to the models, and the main findings remain materially unchanged after adjustment for center sites. Please refer to the revised manuscript for the updated outcomes.

Reviewer comment:

7. Multiple testing. Bonferroni correction is claimed but the test family is unspecified, and reported p-values do not appear corrected? Both uncorrected and corrected p-values should be shown.

Reply:

Thank you very much. We have followed the advice from the reviewers and have now provided raw p-values as well as the uncorrected and corrected values.

Reviewer comment:

8. The ACC/LTP/cytokine narrative in the Discussion is attached to a study that measured none of these. Given that IMAGEN has neuroimaging, the paper would be significantly stronger if imaging data were used.

Reply:

We greatly appreciate this suggestion. We consider neuroimaging data as a future direction, and we have now clarified these points in the manuscript.

"Future studies should examine the neurobiological correlates of these genetic effects using neuroimaging to assess structural and functional connectivity in brain regions such as the ACC, insula, amygdala, and hippocampus. Integration with inflammatory biomarkers would also clarify the immune signaling pathways involved. In addition, studies that include repeated pain assessments across adolescence will be important for disentangling the development and persistence of pain symptoms over time. Ultimately, identifying individuals with genetic susceptibility to affect-induced pain may enable personalised prevention strategies – especially in youth at risk for chronic pain and psychiatric disorders."

Reviewer comment:

9. Attrition and selection bias. IMAGEN's full baseline cohort is ~2,000. However, the authors analyze 724 with complete baseline + follow-up + genotype data. No comparison of included vs. excluded participants on key variables (baseline anxiety, depression, SES, site). Differential attrition by affective symptoms is plausible and would introduce bias into the exposure-outcome association in this study.

Reply:

Thank you for this helpful suggestion. We have now included a comparison of included vs. excluded participants on key variables. The details can be found in Table 5 in the revised manuscript:

Table 5. Participants' characteristics

Characteristics	Included Sample	Excluded sample	Statistics
N	724	1461	
Gender (m/f)	312/412	760/701	$\chi^2(1) = 15.076$, p = 1.033e-4
Age BL	14.439 (0.380)	14.424 (0.394)	*t* = -0.839, p = 0.4017
Anxiety BL	1.233 (0.549)	1.243 (0.562)	$\chi^2(3) = 0.305$, p = 0.959
Depression BL	0.928 (1.006)	0.962 (1.033)	$\chi^2(4) = 1.339$, p = 0.8551
Age FU1	16.097 (0.085)	16.101 (0.086) N=495	*t* = 0.831, p = 0.4061

Sum pain score FU1	3.301 (3.308)	3.212 (3.107)<br>N=495	$\chi^2(19) = 21.448$, $p = 0.3126$
Participation rate per center; n (%)	London 81 (11.187)<br>Nottingham 92 (12.707)<br>Dublin 63 (8.702)<br>Berlin 84 (11.602)<br>Hamburg 106 (14.641)<br>Mannheim 96 (13.260)<br>Paris 80 (11.050)<br>Dresden 122 (16.851)	190 (13.004)<br>269 (18.412)<br>170 (11.636)<br>185 (12.663)<br>159 (10.883)<br>169 (11.567)<br>182 (12.457)<br>137 (9.377)	$\chi^2(7) = 45.567$, $p = 1.061e\text{-}07$

Reviewer comment:

10. Finally, that anxiety/depression precede pain in adolescence is already reasonably well established (indeed, the authors cite existing evidence of this). Instead, the novel contribution in this study is the NRF2 finding, which should be framed as such. However, this finding itself rests on a stratified analysis (with no test for effect modification) in a rather modest sample size (N=724; only 135 TG/TT), and which has rather important methodological issues.

Reply:

We thank the reviewer for this feedback. We agree that the novel contribution of the present study lies primarily in the NRF2-related findings and have revised the manuscript accordingly. We would also like to clarify that the primary objective of this study was to investigate whether NRF2 polymorphisms influence the prospective association between anxiety and depressive symptoms at age 14-15 years and pain complaints two years later. Accordingly, the genotype-stratified analyses constitute the primary outcome of interest. To better reflect this focus, the terminology has been revised throughout the manuscript, including in the Abstract, Introduction, Results, and Discussion sections.

Reviewer comment:

Other minor comments

Bolding for emphasis in Results is distracting; italicisation of NRF2 (gene vs. protein) is inconsistent; the CSI pain subscale items and internal consistency should be reported; DAWBA output is ordinal and should not be treated as continuous without acknowledgement.

Reply:

Thank you for the feedback. We agree with the reviewer's points of view and have now carefully addressed them in the manuscript.

Reviewer comment:

In summary, the two most important issues are the absence of a baseline pain measure and the use of stratified rather than interaction analysis. Together these mean the headline claim (that immune dysfunction explains the association between anxiety and depression and later pain) are not properly tested.

Reply:

We once again appreciate the reviewers' thoughtful comments, which have greatly helped us improve this manuscript. We have carefully addressed all points raised, revised and tempered our interpretations where appropriate, and hope that the revised version is now substantially strengthened.

Reviewers' comments:

Reviewer #1 (Remarks to the Author):

I thank the authors for their thorough rebuttal letter. My concerns have been addressed.

Reviewer #2 (Remarks to the Author):

The authors have satisfactorily addressed my concerns.

Reply:

We thank the reviewers for their suggestions, which greatly helped us to improve the manuscript, and we are delighted that their concerns have been sufficiently addressed.

Reviewer #3 (Remarks to the Author):

Thank you for providing this updated manuscript, which I enjoyed reviewing. I thank the authors for the additional analyses which are welcome (negative-control SNP, permutation test, attrition comparison, site adjustment, and reporting of raw p-values). However, one of my principle concerns remains substantively unaddressed, which I feel is important as it is central to the message of this manuscript.

Reply:

Thank you for your thoughtful and constructive comments. We appreciate and are grateful for the opportunity to address your remaining concerns. In response, we have further revised the manuscript carefully and thoroughly.

Reviewer comment:

Stratified analysis vs. test of effect modification: My original comment was that a moderation claim cannot be supported by comparing a significant association in one genotype group against a non-significant one in another (i.e. a stratified analysis as reported here). Although the revision relabels terminology to some extent, it retains the moderation framing while still resting entirely on stratified models (see Table 4, Models 3 and 4), and no interaction term has been fitted.

This is exemplified in the Results section under heading "Moderation of the Anxiety-Pain Association by NRF2 (rs6721961)", which illustrates the issue. The authors describe "stratified regression analyses" and then conclude that the relationship is "genotype-dependent" and "absent in TG/TT carriers". This states a moderation claim which is never tested. The "absent in TG/TT" characterisation is also not supported by the authors' own Table 4, where the TG/TT slope (beta=0.133 [-0.013, 0.278]) is in the same direction, of similar magnitude, and overlaps substantially with the GG slope (beta=0.238 [0.156, 0.320]). This pattern could also be consistent with the smaller subgroup (N=135) being underpowered rather than with an absent effect, and without formally testing an anxiety×genotype interaction term we cannot know. Moreover, the closing statement that rs35652124 was excluded because it "showed no... gene-

phenotype interactions" implies an interaction test for this SNP that does not appear for rs6721961.

Relatedly, the Abstract states that "gene-by-phenotype interactions" were tested, however no interaction tests appear in any model.

(I would also note that the additional permutation and down-sampling analyses, while welcome, test genotype differences in pain level and the robustness of the GG slope respectively. Neither tests whether the slope, which is of primary interest to their hypothesis, differs by genotype.)

The stated objective of the paper is to investigate "whether two common NRF2 polymorphisms (rs6721961 and rs35652124) moderate the association between anxiety and depression symptoms at age 14-15 and pain complaints two years later at age 16–17."

Given the focus on moderation, I would ask the authors to fit and report a formal anxiety×genotype interaction coefficient, to formally test moderation by genotype.

Alternatively, they should remove all effect-modification language ("moderated," "genotype-dependent," "absent in TG/TT," "resilient") and present the result strictly as a stratified descriptive analysis.

Reply:

Thank you for the helpful feedback, and we apologize for the misunderstanding and still lacking some clearer terminology.

We have now adjusted the manuscript accordingly to strictly adhere to the corresponding analyses, i.e., the stratified descriptive analysis.

Starting with the Abstract:

"This study investigated whether polymorphisms in the NRF2 gene (rs6721961 and rs35652124) are related to the prospective association between anxiety and depression symptoms at age 14-15 and pain complaints two years later at age 16-17 (N=724).

Methods: Data were drawn from the IMAGEN longitudinal study. Anxiety and depression symptoms were assessed using the Development and Well-Being Assessment (DAWBA), and pain complaints were measured via the Children's Somatization Inventory (CSI). Genotyping was conducted for NRF2 SNPs rs6721961 and rs35652124. Regression analyses tested main effects, followed by genotype-stratified analyses."

Then with the Introduction:

"We investigated whether two common NRF2 polymorphisms (rs6721961 and rs35652124) are related to the prospective association between anxiety and depression symptoms at age 14-15 and pain complaints two years later at age 16–17. Specifically, we were interested in whether the genotype groups differ in this association. Based on previous literature, we expected higher internalising symptoms to be associated with greater pain complaints at follow-up and hypothesized that NRF2 *genetic variation would be related to individual differences in susceptibility to this affect-pain relationship."*

And in the Result:

"Anxiety-Pain Association Stratified by NRF2 (rs6721961) Genotype"

"To assess whether the prospective association between early anxiety symptoms and later pain complaints differed across rs6721961 genotype groups, stratified regression analyses were performed:"

...

"These findings suggest potential differences in the relationship between early anxiety and later pain outcomes across rs6721961 genotypes. The predictive effect of anxiety appears to be specific to adolescents with the GG genotype of rs6721961, while it is non-significant among TG/TT carriers (Figure 2)."

"As rs35652124 showed no main effects or differences across genotype groups, it was excluded from further stratified analyses."

Finally, in the Discussion:

"Specifically, carriers of the rs6721961 GG genotype exhibited higher anxiety levels and more pain complaints, and the prospective association between anxiety and pain was significant in this genotype group. In contrast, no significant association between anxiety and later pain outcomes was found in carriers of the TG/TT genotypes."

...

"Together, these findings point to the NRF2 rs6721961 polymorphism as a potential genetic factor that is related to the relationship between early anxiety and later pain. While exploratory, these results contribute to emerging efforts to identify genetically based susceptibility and resilience factors for pain and affective comorbidity"

...

"In conclusion, the present findings suggest that the NRF2 gene variant rs6721961 is associated with the prospective relationship between internalising symptoms and pain during adolescence. Although these findings should not be interpreted as evidence that rs6721961 significantly moderates the association, they highlight the importance of considering genetic heterogeneity when examining complex relationships between psychological and pain-related outcomes and may provide a basis for future research into underlying mechanisms. In addition, while the explained variance is moderate (7.6%), these results align with growing evidence implicating immune and oxidative stress pathways in both emotional and somatic health outcomes. They also support further investigation of NRF2-related genetic variation as a potential contributor to pain vulnerability and as a candidate pathway for understanding early risk trajectories among youth experiencing internalizing symptoms."

Please note that, following the journal's formatting requirements, the order of the Tables has been modified in the revised manuscript.

Reviewer comment:

Finally, regarding reverse causation: I accept the authors explanation that baseline pain was not assessed in IMAGEN and therefore cannot be adjusted for. My remaining concern is therefore one of framing rather than analysis as the causal framing has not been brought into line with this limitation. Phrases such as "affect-induced pain", "confer greater vulnerability", "developmental trajectory from early anxiety to later pain", and "target for early intervention" remain throughout. Given that pre-existing pain could plausibly elevate baseline anxiety and track forward to follow-up pain, I would ask that this limitation be foregrounded as a primary limitation and that causal language be removed rather than balanced against a later caveat in the discussion.

Reply:

Thank you for this suggestion. We totally agree with this and have now adjusted the limitations accordingly as well as make corresponding changes regarding the causal language.

"Several limitations should be acknowledged. First, the absence of baseline pain assessment within the IMAGEN dataset at age 14 restricted our ability to determine the extent to which the observed affect-pain association is independent of pre-existing pain symptoms. This should

be investigated in future studies. Second, while our sample was large and longitudinal, genotype frequencies limited the statistical power for certain subgroup analyses. Pain and affective symptoms were self-reported, and clinical diagnoses or biomarkers (e.g., cytokine levels) would strengthen the findings. We focused on two NRF2 SNPs; other polymorphisms or epigenetic factors may also play a role. Additionally, limitations inherent to candidate gene studies, such as multiple testing issues and low reproducibility, should not be overlooked. Finally, adjusting the regression models for other pain-sensitive factors, such as childhood adversity and sleep, may further improve the models."

Reviewers' comments:

Reviewer #3 (Remarks to the Author):

Reviewer comment:

Thank you for providing this revised manuscript and for your careful response to my comments on the previous draft. I am satisfied that my principal concerns have now been substantially addressed, particularly through reframing the genotype analyses as stratified rather than as evidence of formal moderation, and by appropriately foregrounding the absence of baseline pain as a major limitation.

Reply:

Thank you very much for your positive comments. We are pleased that our revisions have addressed your principal concerns, and we are grateful for the opportunity to further clarify the remaining points.

Reviewer comment:

I would, however, suggest one final tightening of the language around the genotype findings: given that no formal anxiety-by-genotype interaction has been demonstrated, statements that the association appears "specific" to GG carriers or that the relationship differs between genotype groups should remain explicitly descriptive.

Reply:

Thank you for this important clarification. We apologize for not having been sufficiently precise in the previous version. We have now carefully revised the manuscript throughout to ensure that the genotype findings are presented consistently in explicitly descriptive terms and do not imply a formal moderation effect.

For example, the following sentence in the Results section:

"*The predictive effect of anxiety appears to be specific to adolescents with the GG genotype of rs6721961, while it is non-significant among TG/TT carriers (Figure 2)*."

has been revised to become explicitly descriptive:

"*The association was significant among adolescents with the GG genotype of rs6721961, but not among TG/TT carriers (Figure 2).*"

This revised wording is intended to describe the observed associations within the genotype-stratified analyses without implying that the strength of the associations statistically differs between genotype groups.

Reviewer comment:

The absence of baseline pain also substantially limits the extent to which the temporal association can be interpreted as anxiety preceding the development of pain. Subject to these relatively minor points, I think the manuscript is considerably improved.

Reply:

We appreciate this comment and fully agree with this point. In response, we have removed the term "prospective" when describing this relationship and have revised the wording throughout the manuscript to avoid implying that anxiety preceded the onset or development of pain.

Instead, we now explicitly describe the observed temporal association as the relationship between early internalizing symptoms and later pain during adolescence, or, more specifically, between internalizing symptoms at ages 14–15 and pain at ages 16–17. We believe this wording more accurately reflects the available data, given the absence of a baseline measure of pain.

We hope that these revisions adequately address the reviewer's remaining concerns and further clarify the interpretation of our findings.

" *Consistent with previous literature, we found that greater levels of anxiety and depression at age 14–15 are associated with increased pain complaints at age 16–17. Individuals carrying the TG/TT genotype of rs6721961 report significantly lower anxiety scores and fewer pain complaints than GG genotype carriers. Notably, in the genotype-stratified analyses, the association between early anxiety and later pain reaches statistical significance among individuals with the GG genotype, but not among those with the TG/TT genotype*."
