# Supplementary material for: Immune-related gene expression links anxiety, depression, and pain outcomes in adolescence
Source: Commun Med (Lond). 2026 Oct 16;6:539. doi: 10.1038/s43856-026-01960-5 (PMC13675203; doi:10.1038/s43856-026-01960-5)
Supplement: Supplementary file 2 [file 43856_2026_1960_MOESM2_ESM.pdf]

Supplementary Materials

Table S1. Characteristics of CSI pain-related items for rs2886161

variable	CC	CT	TT	statistic (H)	p value raw	p value adjusted
Pain heart chest	0.293 (0.564)	0.288 (0.532)	0.226 (0.542)	5.140227533	7.652684e-02	3.826342e-01
Sore muscles	0.693 (0.771)	0.615 (0.769)	0.641 (0.743)	1.113659316	5.730229e-01	9.650210e-01
Trouble getting breath	0.333 (0.622)	0.17 (0.474)	0.169 (0.474)	9.251365646	9.796963e-03	9.796963e-02
Numbness or tingling	0.293 (0.514)	0.308 (0.515)	0.323 (0.556)	0.099157027	9.516304e-01	9.650210e-01
Weakness	0.56 (0.721)	0.564 (0.701)	0.496 (0.678)	1.990668212	3.695999e-01	9.650210e-01
Heavy feelings in arms legs	0.36 (0.584)	0.404 (0.664)	0.374 (0.619)	0.139076136	9.328246e-01	9.650210e-01
Heart beating too fast	0.2 (0.493)	0.224 (0.539)	0.172 (0.436)	1.044758522	5.931077e-01	9.650210e-01
Difficulty swallowing	0.173 (0.554)	0.147 (0.414)	0.148 (0.41)	0.160113565	9.230639e-01	9.650210e-01
Pain knees elbows joints	0.267 (0.553)	0.337 (0.594)	0.353 (0.634)	1.388783269	4.993782e-01	9.650210e-01
Pain arms legs	0.333 (0.644)	0.288 (0.532)	0.312 (0.588)	0.071210891	9.650210e-01	9.650210e-01
Pain sum score	3.507 (3.696)	3.346 (3.271)	3.214 (3.258)	0.439114477	8.028742e-01	8.028742e-01

Table S2. Anxiety, Depression, and rs2886161

rs2886161	Anxiety	Depression	N
CC	1.2 (0.465)	0.987 (0.908)	75
CT	1.228 (0.516)	0.910 (1.013)	312
TT	1.246 (0.594)	0.932 (1.026)	337
	p = 0.970837512	p = 0.501277676	
Data is presented as mean (SD)			

Table S3. Regression analysis after random downsampling of the GG rs6721961

term	Model total sample	Model downsample
(Intercept)	0.226	0.616
	SE = 0.129	SE = 0.325
	p = 0.081	p = 0.060
	95% CI [-0.028, 0.479]	95% CI [-0.027, 1.259]
GAD BL	0.238	0.511
	SE = 0.042	SE = 0.102
	p = 1.87e-08	p = 1.81e-06
	95% CI [0.156, 0.320]	95% CI [0.309, 0.713]

sex	-0.176	0.229
	SE = 0.086	SE = 0.200
	p = 0.0425	p = 0.255
	95% CI [-0.345, -0.006]	95% CI [-0.167, 0.626]
centre_id2	-0.025	-0.350
	SE = 0.167	SE = 0.429
	p = 0.880	p = 0.416
	95% CI [-0.354, 0.304]	95% CI [-1.199, 0.499]
centre_id3	-0.023	-0.272
	SE = 0.187	SE = 0.446
	p = 0.904	p = 0.543
	95% CI [-0.391, 0.346]	95% CI [-1.154, 0.610]
centre_id4	-0.047	-0.910
	SE = 0.172	SE = 0.410
	p = 0.785	p = 0.0284
	95% CI [-0.386, 0.292]	95% CI [-1.723, -0.098]
centre_id5	-0.001	-0.487
	SE = 0.164	SE = 0.386
	p = 0.997	p = 0.209
	95% CI [-0.322, 0.321]	95% CI [-1.251, 0.277]
centre_id6	-0.222	-0.781
	SE = 0.168	SE = 0.406
	p = 0.188	p = 0.057
	95% CI [-0.553, 0.109]	95% CI [-1.585, 0.024]
centre_id7	-0.055	-0.558
	SE = 0.173	SE = 0.416
	p = 0.750	p = 0.182
	95% CI [-0.395, 0.285]	95% CI [-1.381, 0.264]
centre_id8	-0.379	-0.997
	SE = 0.164	SE = 0.390
	p = 0.0208	p = 0.0118
	95% CI [-0.700, -0.058]	95% CI [-1.770, -0.224]
N	589	135
Adjusted R^2	0.076	0.185
	1.17E-08	5.67E-05
pain sum score_FU1 ~ GAD BL + sex + centre_id		

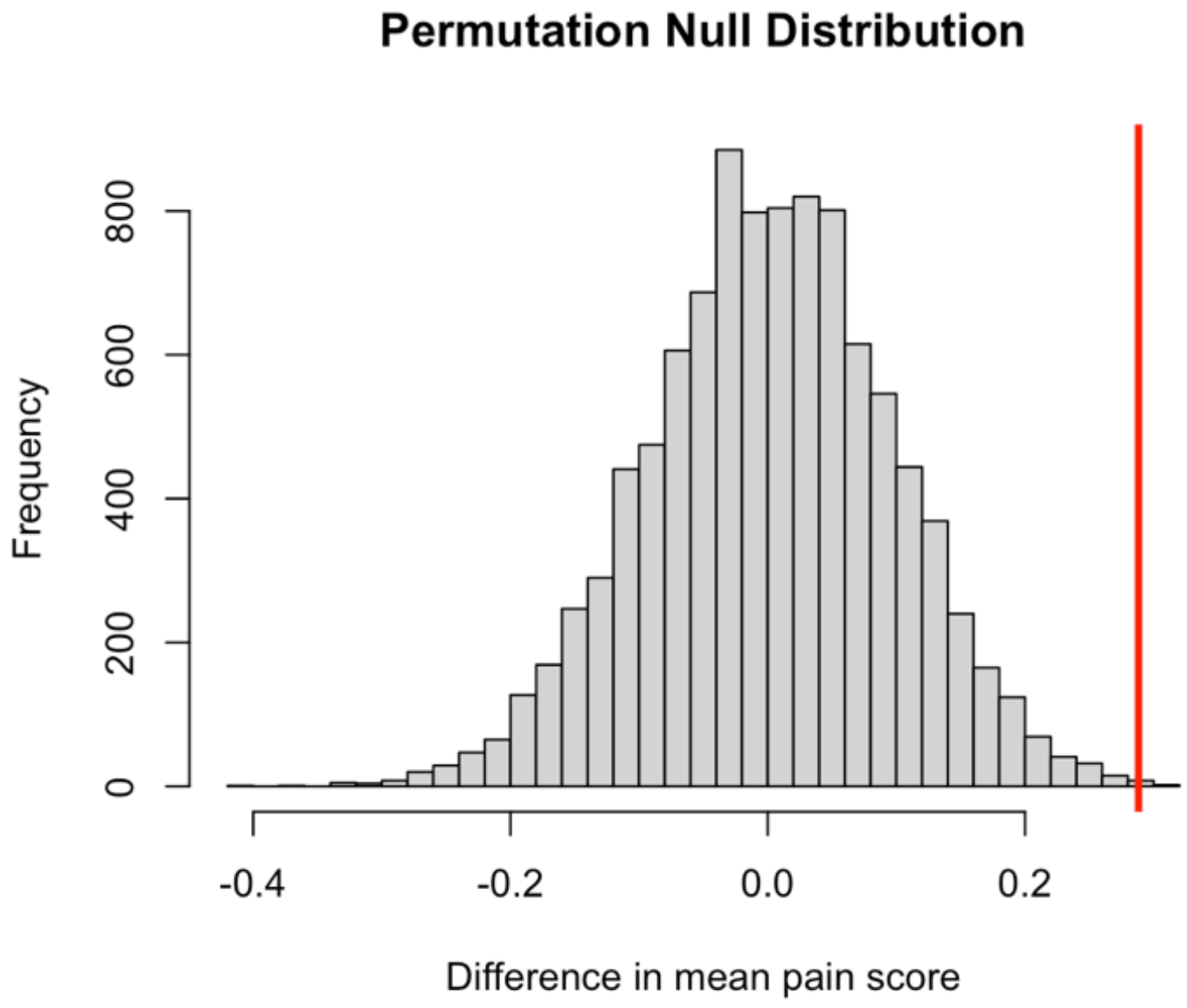

Figure S1. Permutation test for the difference in mean pain sum score between rs6721961 genotypes. A permutation test (10000 permutations) showed that the observed difference in mean pain sum score between rs6721961 genotypes was unlikely under the null distribution (empirical p = 0.0022). The red line represents the observed difference.
