# Supplementary material for: Immune-related gene expression links anxiety, depression, and pain outcomes in adolescence
Source: Commun Med (Lond). 2026 Oct 16;6:539. doi: 10.1038/s43856-026-01960-5 (PMC13675203; doi:10.1038/s43856-026-01960-5)
Supplement: Supplementary file 3 [file 43856_2026_1960_MOESM3_ESM.pdf]

Description of Additional Supplementary Files

File name: Supplementary Data 1

Description: Source Data for Figure 2
