# Supplementary material for: Immune-related gene expression links anxiety, depression, and pain outcomes in adolescence
Source: Commun Med (Lond). 2026 Oct 16;6:539. doi: 10.1038/s43856-026-01960-5 (PMC13675203; doi:10.1038/s43856-026-01960-5)
Supplement: Supplementary file 4 [file 43856_2026_1960_MOESM4_ESM.pdf]

Table 6. Summary of regression models

Variable	Model 1	Model 2	Model 3	Model 4
(Intercept)	0.190	0.168	0.226	0.015
	SE = 0.112	SE = 0.112	SE = 0.129	SE = 0.193
	p = 0.08897	p = 0.133	p = 0.081	p = 0.937
	95% CI [-0.029, 0.410]	95% CI [-0.051, 0.387]	95% CI [-0.028, 0.479]	95% CI [-0.367, 0.398]
GAD	0.231		0.238	0.133
	SE = 0.037		SE = 0.042	SE = 0.074
	p = 6.26e-10		p = 1.87e-08	p = 0.0741
	95% CI [0.159, 0.303]		95% CI [0.156, 0.320]	95% CI [-0.013, 0.278]
sex	-0.185	-0.197	-0.176	-0.237
	SE = 0.074	SE = 0.073	SE = 0.086	SE = 0.127
	p = 0.01265	p = 0.00737	p = 0.043	p = 0.063
	95% CI [-0.331, -0.040]	95% CI [-0.341, -0.053]	95% CI [-0.345, -0.006]	95% CI [-0.488, 0.013]
centre_id 2	-0.035	-0.017	-0.025	-0.177
	SE = 0.146	SE = 0.146	SE = 0.167	SE = 0.270
	p = 0.811	p = 0.908	p = 0.880	p = 0.514
	95% CI [-0.322, 0.252]	95% CI [-0.303, 0.270]	95% CI [-0.354, 0.304]	95% CI [-0.710, 0.357]
centre_id 3	-0.009	0.029	-0.023	0.029
	SE = 0.162	SE = 0.161	SE = 0.187	SE = 0.275
	p = 0.954	p = 0.857	p = 0.904	p = 0.916
	95% CI [-0.326, 0.308]	95% CI [-0.287, 0.345]	95% CI [-0.391, 0.346]	95% CI [-0.516, 0.574]
centre_id 4	-0.110	-0.066	-0.047	-0.402
	SE = 0.150	SE = 0.149	SE = 0.172	SE = 0.263

	p = 0.464	p = 0.656	p = 0.785	p = 0.129
	95% CI [-0.403, 0.184]	95% CI [-0.359, 0.226]	95% CI [-0.386, 0.292]	95% CI [-0.923, 0.119]
centre_id 5	-0.022	0.017	-0.001	-0.126
	SE = 0.142	SE = 0.141	SE = 0.164	SE = 0.245
	p = 0.876	p = 0.904	p = 0.997	p = 0.609
	95% CI [-0.300, 0.256]	95% CI [-0.260, 0.295]	95% CI [-0.322, 0.321]	95% CI [-0.611, 0.360]
centre_id 6	-0.164	-0.120	-0.222	0.110
	SE = 0.145	SE = 0.145	SE = 0.168	SE = 0.248
	p = 0.259	p = 0.409	p = 0.188	p = 0.658
	95% CI [-0.449, 0.121]	95% CI [-0.405, 0.165]	95% CI [-0.553, 0.109]	95% CI [-0.380, 0.600]
centre_id 7	-0.035	-0.066	-0.055	0.003
	SE = 0.152	SE = 0.151	SE = 0.173	SE = 0.281
	p = 0.818	p = 0.663	p = 0.750	p = 0.992
	95% CI [-0.333, 0.263]	95% CI [-0.362, 0.230]	95% CI [-0.395, 0.285]	95% CI [-0.554, 0.560]
centre_id 8	-0.378	-0.327	-0.379	-0.285
	SE = 0.138	SE = 0.138	SE = 0.164	SE = 0.221
	p = 0.00625	p = 0.01785	p = 0.0208	p = 0.199
	95% CI [-0.648, -0.107]	95% CI [-0.597, -0.057]	95% CI [-0.700, -0.058]	95% CI [-0.722, 0.152]
MDD		0.247		
		SE = 0.036		
		p = 2.86e-11		
		95% CI [0.175, 0.318]		
N	724	724	589	135

Adjusted R^2	0.079	0.086	0.076	0.053
p value	4.10e-11	2.65e-12	1.17e-08	0.06881

Model1 <- pain sum score (FU1) ~ GAD (BL) + sex + centre_id

Model2 <- pain sum score (FU1) ~ MDD (BL) + sex + centre_id

Stratified regression:

rs6721961 GG

Model3 <- pain sum score (FU1) ~ GAD (BL) +sex + centre_id

rs6721961 TG/TT

Model4 <- pain sum score (FU1) ~ GAD (BL) + sex + centre_id

centre_id1: London

centre_id2: Nottingham

centre_id3: Dublin

centre_id4: Berlin

centre_id5: Hamburg

centre_id6: Mannheim

centre_id7: Paris

centre_id8: Dresden
